# Structural implications of Ca^2+^-dependent actin-bundling function of human EFhd2/Swiprosin-1

**DOI:** 10.1038/srep39095

**Published:** 2016-12-15

**Authors:** Kyoung Ryoung Park, Min-Sung Kwon, Jun Yop An, Jung-Gyu Lee, Hyung-Seop Youn, Youngjin Lee, Jung Youn Kang, Tae Gyun Kim, Jia Jia Lim, Jeong Soon Park, Sung Haeng Lee, Woo Keun Song, Hae-Kap Cheong, Chang-Duk Jun, Soo Hyun Eom

**Affiliations:** 1 School of Life Science, Gwangju Institute of Science and Technology (GIST), 123 Cheomdangwagi-ro, Buk-gu, Gwangju 61005, Republic of Korea; 2 Steitz Center for Structural Biology and Department of Chemistry, Gwangju Institute of Science and Technology (GIST), 123 Cheomdangwagi-ro, Buk-gu, Gwangju 61005, Republic of Korea; 3 Protein Structure Group, Korea Basic Science Institute, 162 Yeongudanji-Ro, Ochang 363-883, Republic of Korea; 4 Department of Cellular and Molecular Medicine, Chosun University School of Medicine, 375 Seosuk-dong, Dong-gu, Gwangju 501-759, Republic of Korea

## Abstract

EFhd2/Swiprosin-1 is a cytoskeletal Ca^2+^-binding protein implicated in Ca^2+^-dependent cell spreading and migration in epithelial cells. EFhd2 domain architecture includes an N-terminal disordered region, a PxxP motif, two EF-hands, a ligand mimic helix and a C-terminal coiled-coil domain. We reported previously that EFhd2 displays F-actin bundling activity in the presence of Ca^2+^ and this activity depends on the coiled-coil domain and direct interaction of the EFhd2 core region. However, the molecular mechanism for the regulation of F-actin binding and bundling by EFhd2 is unknown. Here, the Ca^2+^-bound crystal structure of the EFhd2 core region is presented and structures of mutants defective for Ca^2+^-binding are also described. These structures and biochemical analyses reveal that the F-actin bundling activity of EFhd2 depends on the structural rigidity of F-actin binding sites conferred by binding of the EF-hands to Ca^2+^. In the absence of Ca^2+^, the EFhd2 core region exhibits local conformational flexibility around the EF-hand domain and C-terminal linker, which retains F-actin binding activity but loses the ability to bundle F-actin. In addition, we establish that dimerisation of EFhd2 via the C-terminal coiled-coil domain, which is necessary for F-actin bundling, occurs through the parallel coiled-coil interaction.

More than 100 actin-related proteins exist in eukaryotic cells, and these proteins regulate the transition of actin polymerisation and depolymerisation to form highly complex structures^1^,^2^,^3^. Actin-related proteins are classified according to their specific functions in actin organisation, such as bundling (crosslinking), severing and capping of the actin cytoskeleton^2^,^4^. Higher ordered actin filaments are stabilised by many actin-bundling proteins that contain coiled-coil domains (cortexillin, SCAB1, coronin-1) and rod domains (α-actinin, villin) for self-association, which organise actin filaments into bundles as homodimers arranged in a parallel or antiparallel fashion. In addition, actin organisation activity of several actin-related proteins is controlled by cellular stimuli (Ca^2+^) and signals^5^,^6^,^7^,^8^,^9^.

Intracellular Ca^2+^ levels affect actin organisation in various ways. Several actin-related proteins contain EF-hands or Ca^2+^/CaM binding domains (see Supplementary Fig. S1). For example, caldesmon contains a Ca^2+^/CaM binding domain that is located close to actin-binding sites. At high Ca^2+^ concentrations (>1 μM), Ca^2+^/CaM binds to caldesmon and interferes with the binding of caldesmon to actin^3^,^10^. In addition, fimbrin and non-muscle α-actinin contain multiple calponin-homology (CH) domains and EF-hands. These proteins associate with actin through CH domains, and F-actin binding or bundling activity is inhibited by Ca^2+^ 11. Conformational changes to EF-hands upon Ca^2+^ binding has been postulated to disrupt the interaction between the CH domain and actin, because EF-hands are located proximal to CH domains (see Supplementary Fig. S1)^11^,^12^,^13^,^14^. For example, the structure of Ca^2+^-free EF-hands of non-muscle α-actinin-1 revealed a flexible conformation around the connecting linker between the N-lobe and C-lobe, and binding of Ca^2+^ to EF-hands induced structural rigidification, which affected the orientation of adjacent CH domains resulting in inhibition of F-actin crosslinking activity^15^. In some cases, such as gelsolin, villin, fragmin and severin, Ca^2+^ directly affects actin-related functions through binding to multiple actin-binding sites. These proteins show F-actin bundling activity at low Ca^2+^ concentrations (<0.1 μM), but actin filament severing activity at high Ca^2+^ concentrations. For these proteins, multiple actin-binding sites bind to F-actin in an open conformation at high Ca^2+^ concentrations, which leads to a transition from F-actin bundling to severing activity (see Supplementary Fig. S1)^3^,^4^,^11^,^16^.

EFhd2/Swiprosin-1 (EFhd2) is a cytoskeletal Ca^2+^-binding protein identified in human immune, brain and mast cells^17^,^18^,^19^. EFhd2 is highly conserved among homologous EF-hand-containing proteins, including EFhd1/Swiprosin-2 (EFhd1) and allograft inflammatory factor-1 (AIF-1). EFhd2 and EFhd1 consist of a disordered N-terminal region followed by two EF-hands and a coiled-coil domain at the C-terminus (see Supplementary Fig. S2a). Although EFhd2 and EFhd1 have similar predicted domain compositions, except for the disordered N-terminal region, their cellular functions are different. EFhd2 is a cytoskeleton-associated protein involved in regulating immune and brain cell functions, whereas EFhd1 appears to modulate apoptosis and differentiation of neuronal and muscle cells by mitochondrial association^20^,^21^. The domain architecture of AIF-1 is different when compared with EFhd1 and EFhd2 (see Supplementary Fig. S2b). Nevertheless, AIF-1 is an F-actin bundling protein that functions, like EFhd2, to regulate the immune system^20^. Recently, among these homologous proteins, the role of EFhd2 in modulating actin dynamics has been examined. EFhd2 modulates cytokine expression by actin remodelling in human mast cells and functions in cancer invasion and metastasis as an actin-related regulator of membrane dynamics^22^,^23^,^24^,^25^. In our previous work, EFhd2 was found to contain multiple actin-binding sites in the core region, including the proline-rich region (PxxP motif) and two EF-hands^26^. We also reported previously that the EF-hands of EFhd2 are involved directly in F-actin bundling in a Ca^2+^-dependent manner and the coiled-coil domain is essential to the F-actin bundling activity by homodimerisation^26^. However, the detailed molecular mechanism describing F-actin binding and bundling by EFhd2 remains elusive because structural data are missing.

Here, we report crystal structures of the Ca^2+^-bound EFhd2 core domain (_CD_EFhd2, residues 70–184) comprising the N-terminal PxxP motif, two EF-hands, ligand mimic (LM) helix and C-terminal linker. In addition, we also report crystal structures of mutants of _CD_EFhd2 defective for Ca^2+^-binding. Furthermore, we performed chemical shift perturbation (CSP), ensemble refinement and biochemical analyses to further understand the structural basis for the Ca^2+^-dependent F-actin bundling function of EFhd2. Based on the experimental results, we propose that the F-actin bundling activity of EFhd2 depends on structural rigidity conferred by binding of two Ca^2+^ ions to the EF-hand domains. In the absence of Ca^2+^, EFhd2 displays local conformational flexibility around the Ca^2+^-binding loop of the EF-hand and C-terminal linker, supporting an explanation of the Ca^2+^-dependent reorganisation of actin binding sites of EFhd2 that retains F-actin binding activity but loses the ability to bundle F-actin. Additionally, we established that dimerisation of EFhd2 via the C-terminal coiled-coil domain, which is necessary for F-actin bundling, occurs through the parallel coiled-coil interaction.

## Results

### Crystal structure of _CD_EFhd2 in the Ca^2+^ bound state

**Figure 1:**
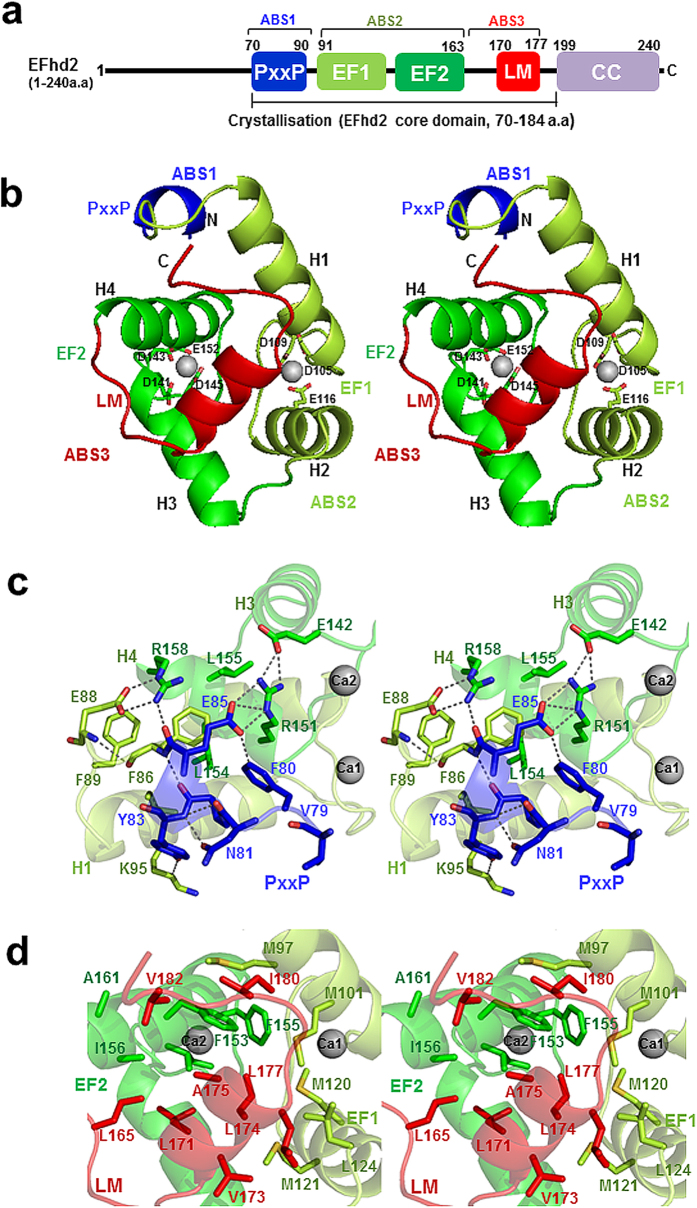
Crystal structure of human _CD_EFhd2. (**a**) Schematic of human _CD_EFhd2; PxxP: proline-rich region, EF: EF-hands, LM: ligand mimic, CC: coiled-coil, ABS: actin-binding site. (**b**) Stereoview of the _CD_EFhd2 structure. The colour coding used is the same as in (**a**). Silver spheres indicate two Ca^2+^ ions located near the loops between the EF-hand motifs. (**c**) Hydrophobic interactions and hydrogen bonds between the PxxP motif and the EF-hand domain. Phe80, Pro82, Phe86 and Phe89 in the PxxP motif are proximal to Phe150, Leu154 and Leu155 in helix 4. Tyr83, Glu85 and Glu88 in the PxxP motif form hydrogen bonds with Lys95 in helix 1 (H1), and Arg151 and Arg158 in helix 4 (H4). (**d**) Intramolecular interactions between hydrophobic residues of the EF-hand domains and the LM-helix.

The crystal structure of Ca^2+^-bound _CD_EFhd2 (residues 70–184) was solved using the multi-wavelength anomalous dispersion (MAD) method and refined to a *R*_work_ = 16.7% and *R*_free_ = 20.2% at 1.85 Å resolution (Table 1). The structure of _CD_EFhd2 adopts a compact and globular fold composed of the predicted PxxP motif (residues 80–90, actin-binding site 1(ABS1)) at the N-terminus followed by two EF-hands (residues 91–163, actin-binding site 2 (ABS2)), a connecting short α-helix and a C-terminal linker (residues 164–184, actin-binding site 3 (ABS3)) (Fig. 1a,b). The connecting short α-helix of _CD_EFhd2 resembles the ligand helix of EF-hand proteins. Thus, we named this helix the LM-helix^27^. In addition, two Ca^2+^ ions are coordinated by several negative charged residues (Asp105, Asp109 and Glu116 for EF1; Asp141, Asp143, Asp145 and Glu152 for EF2), which are well-known Ca^2+^-coordinating residues in EF-hand domains (Fig. 1b).

The PxxP motif is involved in proper intracellular localisation of target proteins through basic motifs (Arg/Lys), exposed hydrophobic residues and a pair of Pro residues^28^. These three conserved elements of the PxxP motif are important for phosphoinositide binding, penetration of the lipid bilayer and SH3 domain binding, respectively^28^. Interestingly, the PxxP motif of _CD_EFhd2 is not only required for association with the B-cell membrane, but was also identified as part of the multiple actin-binding sites^20^,^26^. Although 10 residues (residues 70–79) of the PxxP motif were disordered in the crystal structure, Pro80, Pro82, Phe86 and Phe89 face towards helix 4 of the EF-hands to form hydrophobic interactions, and Glu85, Glu88 and Tyr83 form hydrogen bonds with Lys95, Arg151 and Arg158 of helix 1 and 4 of the EF-hand domains. As a result, the PxxP motif (ABS1) interacts tightly with the EF-hand domains (ABS2) (Fig. 1c). Furthermore, the EF-hand domains (ABS2) not only interact tightly with the PxxP motif (ABS1) via helix 1 and 4, but also associate with the LM-helix (ABS3) through intramolecular interactions that resemble the intermolecular interactions of Ca^2+^-calmodulin (CaM)-peptide complexes (Fig. 1a,d).

### Structural implications of EFhd2 in the absence of Ca^2+^

**Figure 2:**
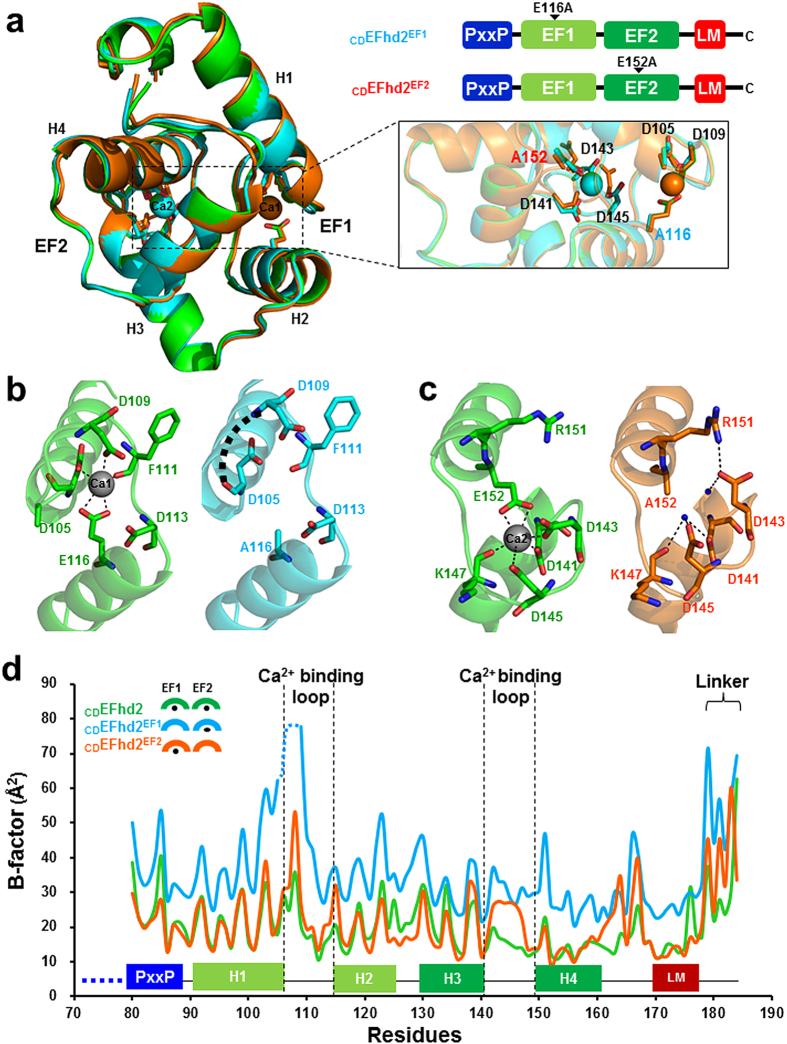
Structure comparison between Ca^2+^-bound _CD_EFhd2 (green), _CD_EFhd2^EF1^ (cyan) and _CD_EFhd2^EF2^ (orange). (**a**) Structural superposition of Ca^2+^-bound _CD_EFhd2 (green), _CD_EFhd2^EF1^ (cyan), and _CD_EFhd2^EF2^ (orange). (**b**) The Ca^2+^-binding loop of EF1 of Ca^2+^-bound _CD_EFhd2 (green) and _CD_EFhd2^EF1^ (cyan). (**c**) Ca^2+^-binding loop of EF2 of Ca^2+^-bound _CD_EFhd2 (green) and _CD_EFhd2^EF2^ (orange). (**d**) Plot of the crystallographic B-factor per residue of Ca^2+^-bound _CD_EFhd2 (20.4 Å^2^, green), _CD_EFhd2^EF1^ (35.6 Å^2^, cyan) and _CD_EFhd2^EF2^ (21.5 Å^2^, orange). The disordered region of the PxxP motif in the crystal structure of _CD_EFhd2 is shown by the blue dashed line.

We failed to determine the structure of the apo form of EFhd2 owing to structural instability during protein purification; however, we could determine the crystal structures of _CD_EFhd2 mutants defective for one Ca^2+^-binding site (E116A for EF1, _CD_EFhd2^EF1^; E152A for EF2, _CD_EFhd2^EF2^) (see Supplementary Fig. S3). The overall structures of these two mutants in the presence of Ca^2+^ are similar to that of Ca^2+^-bound _CD_EFhd2 (_CD_EFhd2^EF1^, root mean square deviation (RMSD) = 0.34 Å for 102 Cα atoms; _CD_EFhd2^EF2^, RMSD = 0.61 Å for 105 Cα atoms), which implies a single Ca^2+^-loaded EF-hand is sufficient to maintain a stable fold (Fig. 2a). However, the Ca^2+^-binding loop region of EF1 of _CD_EFhd2^EF1^ (Arg106, Gly107 and Arg108) was observed to be disordered (Fig. 2b). In addition, in the structure of _CD_EFhd2^EF2^, one water molecule occupied the Ca^2+^ position of EF2 and forms several hydrogen bonds with Asp141, Asp143, Asp145 and Lys147. Moreover, Asp143 forms a hydrogen bond with Arg151 and the Ca^2+^-binding loop is slightly shifted (~2.2 Å). As a result, the water molecule is trapped in the Ca^2+^-binding site of EF2 (Fig. 2c). Furthermore, comparison of the crystallographic B-factors between Ca^2+^-bound and EF-hand mutant structures showed that the largest changes in B-factor values were for _CD_EFhd2^EF1^ (35.6 Å^2^), _CD_EFhd2 (20.4 Å^2^) and _CD_EFhd2^EF2^ (21.5 Å^2^). In particular, B-factor values for EF1 and the C-terminal linker region in the structure of _CD_EFhd2^EF1^ were increased significantly (Fig. 2d). On the other hand, only small changes of B-factor values for EF2 in the structure of _CD_EFhd2^EF2^ were observed and are probably because of small structural perturbations in the absence of Ca^2+^ and stabilisation by newly formed hydrogen bonds to the trapped water molecule. These results suggest that the Ca^2+^-binding loop of EF1 adopts a more flexible structure than EF2 in the absence of Ca^2+^, resulting in large conformational fluctuations to EF1 and a concomitant increase in the overall B-factor. Next, we performed CSP analysis using the conditions of Ca^2+^-free and bound states to identify conformational changes to _CD_EFhd2 in the absence Ca^2+^. The Ca^2+^-dependent conformational changes to _CD_EFhd2 were monitored by measuring resonance perturbations in 2D ^1^H-^15^N HSQC spectra (see Supplementary Fig. S4a). Consistent with the crystal structures of the _CD_EFhd2 mutant, significant CSPs were associated with residues in Ca^2+^ binding loop region (Phe101, Asp105, Asp109, Phe111, Ile 112 and Glu116) of EF1. Noticeably, CSPs of hydrophobic residues in the Ca^2+^-binding loop of EF1 are likely to be associated with the failure of structure determination caused by the instability of EFhd2 in the absence of Ca^2+^. Significant CSPs for the disordered region (residues 70–80, PxxP motif) of the crystal structure appears to be associated with the conformational changes to EF1 in the absence of Ca^2+^. On the other hand, noticeable CSPs were not detected in the LM-helix region (see Supplementary Fig. S4b,c). Thus, we expect the LM-helix region to maintain its structure in the absence of Ca^2+^.

Collectively, we postulate that Ca^2+^ depletion leads to local conformational flexibility of actin-binding sites (EF1, C-terminal linker) and this reduces the F-actin bundling ability of EFhd2 in the absence of Ca^2+^, as observed in previous results^26^.

### Ensemble refinement of the _CD_EFhd2, _CD_EFhd2^EF1^ and _CD_EFhd2^EF2^

**Figure 3:**
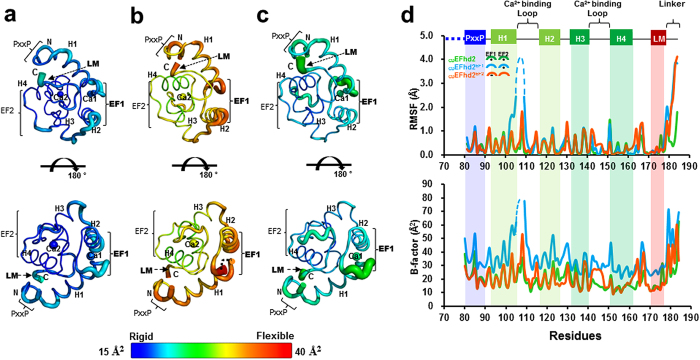
Structural comparison of ensemble models of _CD_EFhd2, _CD_EFhd2^EF1^ and _CD_EFhd2^EF2^. Ensemble models of (**a**) Ca^2+^-bound _CD_EFhd2, (**b**) _CD_EFhd2^EF1^ and (**c**) _CD_EFhd2^EF2^. Structures coloured by B-factor. The B-factors are coloured from a blue/thin line to a red/thicker line with increasing B-factor. **(d)** The root-mean-square fluctuation (RMSF) of ensemble models for Ca^2+^-bound _CD_EFhd2 (green), _CD_EFhd2^EF1^ (cyan) and _CD_EFhd2^EF2^ (orange). RMSF in the EF1 of _CD_EFhd2^EF1^ is consistent with the crystallographic B-factor. The disordered region of the PxxP motif in the crystal structure of _CD_EFhd2 is shown by the blue dashed line.

Based on the results of the crystal structures and CSP analysis, we hypothesise that Ca^2+^ depletion induces changes in local flexibility of the actin binding sites of EFhd2, which implies a Ca^2+^-dependent regulation of F-actin bundling activity of EFhd2 through protein dynamics. To evaluate the effect of Ca^2+^ on structural dynamics of EFhd2 at the atomic level, we performed ensemble refinement for _CD_EFhd2, _CD_EFhd2^EF1^ and _CD_EFhd2^EF2^ using Phenix.ensemble refinement^29^. Ensemble refinement is a useful tool to highlight functional protein dynamics through X-ray diffraction data^29^. Ensemble refinement of the _CD_EFhd2, _CD_EFhd2^EF1^ and _CD_EFhd2^EF2^ yielded a large number of models that represent structural dynamics and decreases in the *R*_free_ value (_CD_EFhd2 = 5.2%, _CD_EFhd2^EF1^ = 3.7%, _CD_EFhd2^EF1^ = 3.8%) compared with the single structure. In the model structures, different degrees of mobility in _CD_EFhd2, _CD_EFhd2^EF1^ and _CD_EFhd2^EF2^ were observed (Fig. 3). As expected, Ca^2+^-bound _CD_EFhd2 displayed a rigid conformation and _CD_EFhd2^EF1^ showed the largest degrees of mobility, indicating that Ca^2+^ depletion of EF1 has a larger impact on conformational dynamics (Fig. 3a,b). In addition, _CD_EFhd2^EF2^ also showed a moderate degree of mobility (Fig. 3c). The significant increase of the root-mean-square fluctuation (RMSF) in the EF1 of _CD_EFhd2^EF1^ is consistent with the crystallographic B-factor and CSP analysis, which support an increase in local flexibility of the actin binding sites of EFhd2 in the absence of Ca^2+^ (Fig. 3d). Interestingly, the C-terminal linker (residues, 176–184) followed by the LM-helix also showed significant RMSF increases in _CD_EFhd2^EF1^ and _CD_EFhd2^EF2^ (Fig. 3d). These results support the postulate that the EFhd2 core domain forms local dynamic conformations (EF1, C-terminal linker) in the absence of Ca^2+^.

### High Ca^2+^ binding affinities of two EF-hand domains

**Figure 4:**
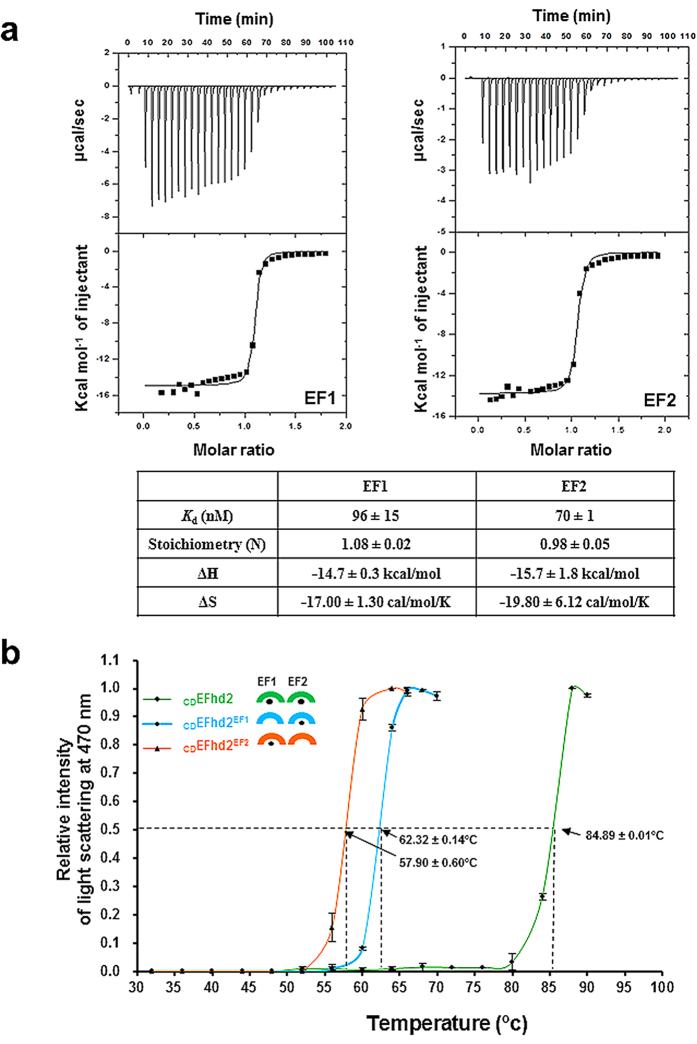
Ca^2+^-binding properties of EFhd2. (**a**) ITC data for EFhd2 with Ca^2+^. Each experiment was repeated in triplicate. (**b**) Light scattering intensity at 470 nm and various temperatures for thermal aggregation of _CD_EFhd2 (green), _CD_EFhd2^EF1^ (cyan) and _CD_EFhd2^EF2^ (orange). The protein concentration was 250 μM. Each experiment was repeated in triplicate. A reduction in intensity at high temperatures indicates precipitation of large-sized aggregates^70^.

In order to measure the Ca^2+^-binding affinity of each EF-hand domain, we performed isothermal titration calorimetry (ITC) experiments using full-length EFhd2^EF1^ and EFhd2^EF2^. We observed that each EF-hand domain possesses high Ca^2+^ binding (EF1, *K*_d_ = 96 ± 15 nM; EF2, *K*_d_ = 70 ± 1 nM). The ITC results for Ca^2+^ binding to EFhd2 mutants (EFhd2^EF1^, EFhd2^EF2^) demonstrate exothermic profiles; thus, the reaction is enthalpically driven. For the EF1 site (using EFhd2^EF2^), *K*_d_ = 96 ± 15 nM, stoichiometry = 1.08 ± 0.02, ΔH = −14.7 ± 0.3 kcal/mol, and ΔS = −17.00 ± 1.30 cal/mol/K. For the EF2 site (EFhd2^EF1^), *K*_d_ = 70 ± 1 nM, stoichiometry = 0.98 ± 0.05, ΔH = −15.7 ± 1.8 kcal/mol, and ΔS = −19.80 ± 6.12 cal/mol/K (Fig. 4a). The decrease in entropy upon Ca^2+^ binding indicates that the flexible conformation of the Ca^2+^ binding site in the absence of Ca^2+^ changes to a rigid conformation. The Ca^2+^-binding affinity of EF-hand containing proteins is related to protein stability. In other words, high affinity towards Ca^2+^ leads to instability in the Ca^2+^-free state^30^. To assess the effect of Ca^2+^ in EFhd2 stability, we measured the Ca^2+^-dependent EFhd2 thermostability thorough a heat aggregation test (Fig. 4b). Consistent with a previous study showing that thermostability of EFhd2 was restored by Ca^2+^ at a high temperature^31^, the half aggregation temperature for both EF-hand mutants that bind only one Ca^2+^ is significantly lower (_CD_EFhd2^EF1^: 62.32 ± 0.14 °C, _CD_EFhd2^EF2^: 57.90 ± 0.60 °C) than the two Ca^2+^-bound EFhd2 (_CD_EFhd2: 84.89 ± 0.01 °C) and is consistent with ref. 30 and 31.

Although the crystal structures of EF-hand mutants (_CD_EFhd2^EF1^ and _CD_EFhd2^EF2^) are similar to Ca^2+^-bound _CD_EFhd2, we observed structural flexibility in the Ca^2+^-binding loop of EF1 and the C-terminal linker (Figs 2 and 3). Based on structural and biochemical results, we hypothesise that changes in the conformation and flexibility lead to exposure of hydrophobic residues around the Ca^2+^-binding loop of EF-hands and the C-terminal linker, and this exposure of hydrophobic residues affects protein stability.

**Figure 5:**
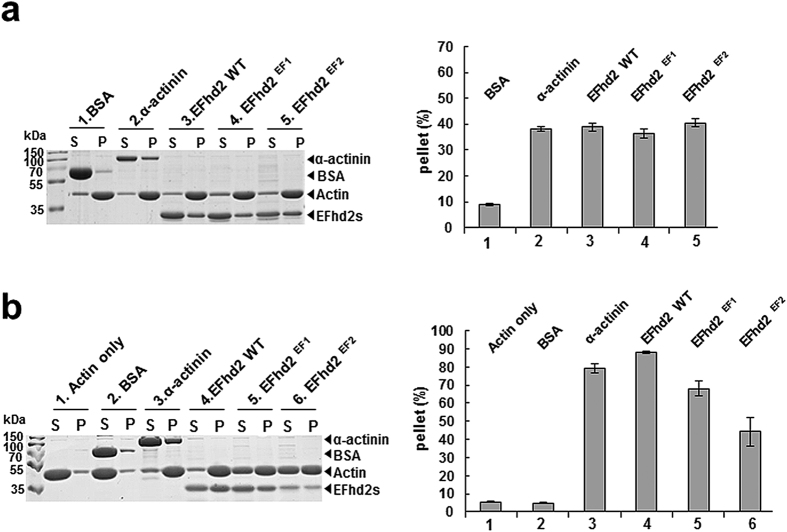
*In vitro* co-sedimentation (F-actin binding) assay and low-speed F-actin bundling assay. (**a**) Co-sedimentation assay and (**b**) low-speed actin-bundling assay of EFhd2 (full-length) and its mutants. Protein samples (5 μM) were added to pre-polymerised actin (2 μM) in the presence of 1 mM CaCl_2_. BSA and α-actinin were used as a negative and positive control, respectively. The percentage of actin distribution was quantified and is presented in the bar graphs. Each experiment was repeated in triplicate.

We previously reported that F-actin bundling activity decreases in the absence of Ca^2+^ 26. We further analysed the contribution of Ca^2+^ binding to each EF-hand domain on F-actin binding and bundling activity (Fig. 5). It is interesting to note that wild-type (Ca^2+^-bound and Ca^2+^-unbound) and each EF-hand mutant (only one Ca^2+^-bound) showed similar F-actin binding activity. Surprisingly, however, F-actin bundling activities were quite different and dependent on the Ca^2+^-bound states. Even depletion of one Ca^2+^ site in a two Ca^2+^-binding protein showed a dramatic reduction in F-actin bundling activity to a level that is similar to that of previously reported data for a two Ca^2+^-depleted state^26^. We propose that the increased structural flexibility observed in the Ca^2+^-binding loop and C-terminal linker, which encompass actin-binding sites, cause a reduction in F-actin bundling activity, presumably because coordination of the F-actin binding sites for F-actin bundling is disrupted.

### Structural comparison between _CD_EFhd2 and a homologous protein, allograft inflammatory factor-1 (AIF-1)

AIF-1 and EFhd1/EFhd2 are highly evolutionarily conserved proteins, because these genes are generated from common ancestral species of the *Bilateria*^20^. In particular, EFhd2 and AIF-1 exhibit the same cellular function as an actin-binding protein. Although sequence homology between AIF-1 and EFhd2 is limited to the EF-hand domains, AIF-1 exhibits F-actin binding and crosslinking activity similar to that observed for EFhd2 (see Supplementary Fig. S2b). EFhd2 exhibits F-actin bundling activity in a Ca^2+^-dependent manner, whereas AIF-1 does not exhibit Ca^2+^ dependency for F-actin binding and bundling activity^26^,^32^,^33^,^34^.

The structure of AIF-1 has been determined in the presence and absence of Ca^2+^ (PDB IDs: 1WY9 and 2D58)^35^. To investigate the molecular basis of the effect of Ca^2+^ on F-actin bundling activity, we compared Ca^2+^-bound and Ca^2+^-free structures of _CD_EFhd2 and AIF-1. The _CD_EFhd2 structure is similar to the structures of Ca^2+^-bound and the apo form of AIF-1 (1WY9, Ca^2+^-bound form_Z-score = 4.0, RMSD = 2.48 Å for 76 Cα atoms; 2D58, apo-form_Z-score = 5.5, RMSD = 1.98 Å for 86 Cα atoms), even though we failed to solve the Ca^2+^-free structure owing to protein destabilisation during the protein purification process (see Supplementary Fig. S5a). Interestingly, EF1 of AIF-1 is stabilised by hydrogen bonds between Asn60, Asn62 and Asp66 in the absence of Ca^2+^ (see Supplementary Fig. S5b). In addition, a water molecule is trapped in the Ca^2+^ binding site of EF2 in the apo structure of AIF-1 similar to that observed for _CD_EFhd2^EF2^ (see Supplementary Fig. S5c). Therefore, _CD_EFhd2 reveals two Ca^2+^-bound EF-hands, whereas Ca^2+^ bound to only EF2 of AIF-1, because Ca^2+^-binding residues (Asp/Glu) are not conserved in EF1 of AIF-1 (see Supplementary Fig. S2)^35^. These structural features of _CD_EFhd2 and AIF-1 suggest that Ca^2+^ is essential for the formation of a stable structure of _CD_EFhd2, whereas AIF-1 is capable of maintaining a stable structure in the absence of Ca^2+^ through hydrogen bonds involving several residues of the Ca^2+^-binding loop of EF1 and a water molecule located in the Ca^2+^-binding site of EF2. These structural differences between _CD_EFhd2 and AIF-1 support the hypothesis that Ca^2+^ is essential for the actin-bundling function of EFhd2 by maintaining a stable structure, whereas AIF-1 exhibits F-actin binding and bundling activity regardless of Ca^2+^ dependency^32^,^33^,^34^.

### EFhd2 displays an actin-bundling function with the parallel coiled-coil domain at the C-terminus

**Figure 6:**
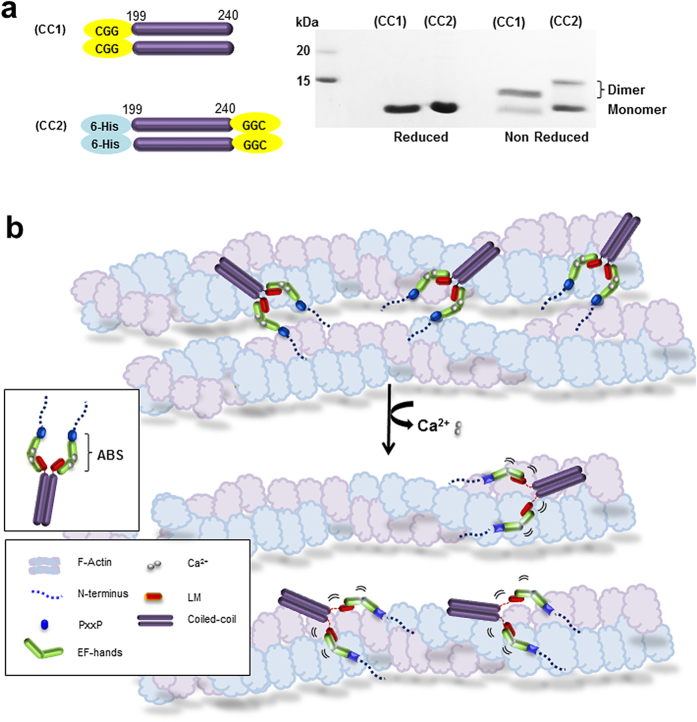
Schematic of the actin-bundling activity of EFhd2. (**a**) SDS-PAGE analysis of the two engineered Cys mutants of the coiled-coil (residues 199–240). Protein samples were analysed under reducing or non-reducing conditions. (**b**) Schematic of the Ca^2+^-dependent F-actin bundling mechanism of EFhd2 (ABS: actin-binding site).

We already reported that the C-terminal coiled-coil domain is essential for the dimerisation of EFhd2 because we observed EFhd2 lost F-actin bundling activity in the absence of the coiled-coil domain^26^. However, it was unclear whether EFhd2 dimerised by parallel or antiparallel interactions. To establish the molecular architecture of EFhd2, we engineered recombinant fragments corresponding to the predicted coiled-coil domain (residues 199–240), including a Cys residue at either the N-terminus (CC1) or C-terminus (CC2) of the coiled-coil domain^36^. We expected that if the coiled-coil domain assembles as a parallel interaction, formation of a disulfide bond should occur owing to the high proximity between Cys residues of each polypeptide and the dimer should be detected on a non-reducing denaturing gel. Therefore, purified recombinant proteins were resolved on sodium dodecyl sulfate polyacrylamide gel electrophoresis (SDS-PAGE) gels under reducing or non-reducing conditions. The disulfide cross-linking assay revealed that SDS-PAGE analysis gave only a monomer band under reducing conditions. In contrast, both CC1 and CC2 protein bands migrated as monomers with the dimeric form observed under non-reducing conditions. These results suggest that the coiled-coil domain of EFhd2 assembles into a parallel dimer (Fig. 6).

## Discussion

Ca^2+^ is an essential modulator of signal transduction processes required for various cellular functions such as contraction, cell differentiation and proliferation^30^. The presence of EF-hand domains in EFhd2 raises the possibility that EF-hand domains upon Ca^2+^ binding may affect its cellular function related to actin dynamics regulation. For example, we found that Ca^2+^ or ethylene glycoltetraacetic acid (EGTA) had little effect on EFhd2 binding to F-actin; however, the F-actin bundling activity was significantly reduced in the Ca^2+^-free state and these results were visualised by electron microscopy^26^.

In this study, we have tried to determine the structure of EFhd2 to elucidate the Ca^2+^-dependent F-actin bundling mechanism of this protein. In a search for structures similar to _CD_EFhd2 using the Dali program^37^, we were able to find ~100 Ca^2+^-bound EF-hands with similar structures (>10 for Z-scores and <3.0 Å in RMSD), in which most of the EF-hand matches were CaM and troponin C (TnC). In addition, the structure of _CD_EFhd2 fits well with those of Ca^2+^-CaM-peptide complexes (see Supplementary Fig. S6)^38^,^39^,^40^,^41^. Ca^2+^-CaM-peptide complexes are more compact than the peptide unbound form because of intermolecular interactions between exposed hydrophobic grooves of CaM and hydrophobic residues of the target molecule. In addition, the Ca^2+^ affinity of EF-hands increases with intermolecular interactions, leading to structural stabilisation of the Ca^2+^-bound state^30^,^42^. Many EF-hand containing proteins can change their diverse biochemical responses through changes in conformation and/or protein stability in the presence or absence of Ca^2+^ 30,42. For example, members of the CaM superfamily are capable of modulating numerous intracellular processes in a Ca^2+^-dependent manner by undergoing conformational changes represented by “close” to “open” structures. On the other hand, several EF-hand containing proteins such as sarcoplasmic Ca^2+^-binding proteins (CaBPs), calcium vector protein (CaVP), calerythrin, and stromal interaction molecule-1 (STIM1) remain in an unstable form in the absence of Ca^2+^ *in vitro*^43^,^44^,^45^,^46^,^47^. In particular, the structure of STIM1 adopts a compact conformation through a hydrophobic interaction between EF-hands and a SAM domain (sterile α motif) similar to Ca^2+^-peptide-CaM complexes and _CD_EFhd2. In addition, mutational analysis revealed that Ca^2+^ depletion or disruption of hydrophobic interactions between EF-hands and the SAM domain leads to destabilisation of the entire EF-SAM complex^43^. Taking into consideration a previous study and structural similarity between Ca^2+^-peptide-CaM complexes, the observations for STIM1 and _CD_EFhd2 support the hypothesis that high affinity for Ca^2+^ and intramolecular interactions of _CD_EFhd2 are likely to maximise stabilisation of the EFhd2 fold. In support of this hypothesis is the thermostability results of EFhd2, which showed that the protein thermal stability at high temperature was restored by Ca^2+^ 31. This is further emphasised by the observation that _CD_EFhd2 remained stable in solution, even at high temperatures in the presence of two Ca^2+^ ions (Fig. 4). As mentioned earlier, in the case of CaM, the core region comprising two EF-hand domains shows significant conformational change upon Ca^2+^ binding, which leads to structural changes in two lobes and interaction with partner proteins. However, in the case of _CD_EFhd2, the structural evidence in this report (including mutant structures and CSP analysis) indicates that the core structure of the EF-hand domains is retained regardless of Ca^2+^ binding, because two hydrophobic clusters in _CD_EFhd2 are maintained (see Supplementary Figure S7a). Denessiouk *et al*. classified EF-hand domains in five groups based on differences in the structural changes in the core region (hydrophobic cluster I and II) upon Ca^2+^ binding^48^. _CD_EFhd2 may belong to type I or IV, because these types have an open conformation in the Ca^2+^-bound form. In the apo state, type I EF-hand domains (Parvalbumin, PVALB) maintain an open conformation; however, type IV EF-hand domains (CaM and Troponin C, TnC) exhibit a closed conformation^48^. This structural difference between types I and IV raised the possibility that the _CD_EFhd2 may belong to type I, because we expect _CD_EFhd2 to have an open conformation in the apo state based on the mutant structures and CSP analysis. Additionally, we compared the structures of the single Ca^2+^-bound state in types I and IV. Intriguingly, in the case of type IV (TnC), the single Ca^2+^-bound intermediate state resembled the closed conformation of the apo state (Supplementary Figure S7b)^49^,^50^,^51^. The structure of the single Ca^2+^-bound state of type I (PVALB) is close to that of the two Ca^2+^-bound state, although the structural difference between the apo and two Ca^2+^-bound states is marginal (Supplementary Figure S7c)^52^,^53^,^54^. This again suggests that _CD_EFhd2 belongs to type I, because the structures of the single Ca^2+^-bound state of the two _CD_EFhd2 mutants are similar to the structure of the two Ca^2+^-bound state, and the core structures of _CD_EFhd2 may not differ even when in a complex with interacting proteins.

We failed to solve the structure of EFhd2 in the absence of Ca^2+^ because of protein instability; however, structures of EF-hand mutants, CSP analysis and ensemble refinement analysis showed that _CD_EFhd2 undergoes changes in local structure and dynamics in the absence of Ca^2+^. The crystal structures of the EF-hand mutants are maintained even when one EF-hand loses Ca^2+^ binding capacity (Fig. 2a). However, the Ca^2+^ binding loop region that loses Ca^2+^ binding activity exhibits structural flexibility (Fig. 2b,d). Furthermore, RMSF values support the premise that F-actin binding sites of EFhd2 form locally dynamic conformations (EF1, C-terminal linker) in the absence of Ca^2+^ and this dynamic state reduces F-actin bundling activity (Figs 3 and 5). In particular, greater flexibility of the C-terminal linker between the EF-hands and coiled-coil domain probably leads to incorrect coordination of actin binding sites in dimer formation. Based on these results, we suggest that the EFhd2 core domain comprising the multiple actin-binding sites changes to an unstable structure by changes in local conformational flexibility in the absence of Ca^2+^, and these structural dynamics reduce the F-actin bundling function.

Recently, a structural model for the Ca^2+^-dependent F-actin crosslinking mechanism by non-muscle α-actinin-1 was reported^15^. Non-muscle α-actinin-1 is composed of N-terminal CH domains (actin binding sites), repeated rod domains and C-terminal EF-hands (CaM-like domain; CaMD). Non-muscle α-actinin-1 forms an antiparallel dimer via the rod domain composed by 4 spectrin-like repeats^11^,^13^. NMR structures of the holo and apo form of CaMD of α-actinin-1 reveal that apo CaMD forms a flexible structure owing to the unstructured linker between N- and C-lobes; however, Ca^2+^ binding leads to stabilisation of the linker, resulting in structural rearrangement of CaMD. Consequently, rearrangement of CaMD inhibits proper orientation of adjacent F-actin binding sites for F-actin crosslinking^15^. This observation supports the concept that Ca^2+^-dependent local conformational flexibility of EFhd2 plays a critical role in regulation of F-actin bundling activity by induced reorganisation of actin-binding sites.

Ca^2+^ is essential for leading edge formation because several Ca^2+^-related actin-binding proteins modulate cell motility and shape by reorganisation of F-actin structures in a Ca^2+^-dependent manner^11^,^55^. For example, F-actin crosslinking activity of non-muscle α-actinin and villin at the leading edge of cells is drastically inhibited at high Ca^2+^ concentrations (micromolar levels)^8^,^9^,^11^. However, Ca^2+^ is required for F-actin bundling function of EFhd2 in contrast with what is observed for α-actinin and villin. We speculate that various Ca^2+^-related F-actin bundling proteins may be involved in F-actin reorganisation as suitable regulators in specific cell environments. Furthermore, in our earlier studies, EFhd2 was mainly expressed at the leading edge of cells and improved lamellipodia formation and cell migration^26^. Interestingly, Beerman *et al*. analysed Ca^2+^ transients of migrating immune cells through direct measurement of Ca^2+^ signalling using light-sheet microscopy. They demonstrated that Ca^2+^ fluctuations were enhanced at the leading edge and reduced at the lagging edge of migrating immune cells^56^. In many EF-hand-containing proteins, including calmodulin, calbindin D9k, and vitamin K-dependent protein S, *K*_d_s for Ca^2+^ are highly dependent on ionic strength. For these proteins, binding affinity for Ca^2+^ is lowered by approximately 2.5–100 fold in the presence of 0.15 M NaCl (close to physiological conditions)^57^,^58^,^59^. We were able to measure *K*_d_s (70–100 nM) for EFhd2 mutants only at low ionic strength (50 mM Tris-HCl, pH 8.5, 20 mM NaCl); we failed to obtain measurements at higher ionic strengths (even at 100 mM NaCl) because of the instability of EFhd2 mutants at higher ionic strengths in the absence of Ca^2+^. Thus, we hypothesise that the affinity of these mutants for Ca^2+^ is much lower than 100 nM, and that both EF hands would not be occupied by Ca^2+^ at resting Ca^2+^ levels in live cells. This result supports the mechanism of cell migration by EFhd2 because Ca^2+^ is essential for the F-actin bundling function of EFhd2.

In conclusion, we demonstrate that EFhd2 shows unique structural and biological features as an EF-hand containing F-actin bundling protein. For F-actin bundling activity, structural stabilisation of the EF-hand domains was found to occur in the presence of Ca^2+^. The core region of EFhd2 maintains its structure in the absence of Ca^2+^ ; however, changes in local conformational flexibility reduce F-actin bundling activity of EFhd2 by incorrect coordination of actin-binding sites in parallel dimer formation. Finally, EFhd2 acts as a cytoskeleton-associated adaptor protein that contains two functional EF-hand domains with high Ca^2+^-binding affinity, which might be a useful target for further research involved in its biological functions or various pathologies^25^,^60^,^61^.

## Methods

### Cloning and protein purification of full-length EFhd2 and ΔNTD

A human EFhd2 clone encoding full-length (residues 1–240) and ΔNTD (residues 70–240) were amplified using the polymerase chain reaction (PCR) from pOTB7 (RZPD German Resource Centre, Germany). Full-length EFhd2 was cloned into a modified pET28a vector (Novagen) containing an N-terminal 6×His (His_6_)-tobacco etch virus (TEV) tag. ΔNTD was cloned into a modified pET28a vector (Novagen) containing a His_6_-Nus-TEV tag. Recombinant DNA were transformed into *E. coli* strain BL21 (DE3) and the cells were grown in Luria–Bertani (LB) medium containing 50 μg/mL kanamycin at 37 °C until the absorbance at 600 nm was 0.7. Expression of recombinant proteins was induced by adding isopropyl β-D-1-thiogalactopyranoside (IPTG) to a final concentration of 0.5 mM and cells were grown for a further 5 h at 37 °C. Cells were harvested by centrifugation (4,000 *g*) for 20 min at 4 °C. The cell pellet was resuspended in buffer containing 50 mM HEPES-NaOH, pH 7.5, 300 mM NaCl and 5 mM imidazole, and the cells disrupted by sonication. After removal of the cell debris by centrifugation at 14,000 *g* for 50 min and 4 °C, the soluble supernatant was loaded onto an equilibrated gravity-flow column (Bio-Rad, Hercules, CA, USA) packed with Ni-NTA agarose resin (Peptron, Korea). The protein was eluted with a buffer containing 50 mM HEPES-NaOH, pH 7.5, 300 mM NaCl and 300 mM imidazole. After concentrating the eluate, the protein solution was incubated with TEV protease overnight at 4 °C to remove the N-terminal His_6_ or His_6_-Nus tag. To exchange the buffer for crystallisation, the final purified protein was passed through a HiLoad 16/60 Superdex 75 gel filtration column (Pharmacia Biotech) pre-equilibrated with 20 mM HEPES-NaOH, pH 7.5, 150 mM NaCl and 1 mM CaC1_2_. Severe degradation was observed after incubation with TEV protease. Therefore, full-length EFhd2 was cloned into a modified pET-21a vector (Novagen) containing N-terminal His_6_ tag. The overall purification procedure was the same as described above. However, the removal process of the N-terminal His_6_ tag was omitted, because the modified pET-21a vector (Novagen) does not include a protease cleavage site.

### EFhd2 core domain (_CD_EFhd2, residues 70–184) identification

We initially tried to crystallise the full-length EFhd2 with ΔNTD. However, crystallisation of this construct failed because of severe degradation during the purification process. Thus, we performed limited proteolysis experiments to identify stable domains and ΔNTD was used for this purpose. Treatment with TEV protease overnight at 4 °C gave a stable fragment, as observed by SDS-PAGE (see Supplementary Fig. S8) and blotted onto a polyvinylidene fluoride membrane to perform N-terminal sequencing analysis (Korea Basic Science Institute, Seoul, Korea). The stable core domain was identified to span residues 70–184 and corresponds to a PxxP motif and two EF-hand domains (see Supplementary Fig. S3).

### Cloning and protein purification of _CD_EFhd2

Human _CD_EFhd2 (residues 70–184) was amplified using PCR from full-length EFhd2 (residues 1–240) and cloned into the modified pET-28a vector (Novagen) containing an N-terminal His_6_-TEV tag. The expressed recombinant protein was purified using the procedure used to purify full-length EFhd2. For seleno-L-methionine (Se-Met) incorporation, a plasmid encoding the _CD_EFhd2 was transformed into the methionine-auxotrophic *E. coli* strain B834 (DE3) (Novagen). Colonies were inoculated into LB medium containing 50 μg/mL kanamycin and incubated at 37 °C with shaking for ~24 h, and then cells were harvested by centrifugation at 4,000 *g* for 20 min and 4 °C. The cell pellet was resuspended in minimal medium to wash and remove the LB medium, and washed cells were harvested by centrifugation at 4,000 *g* for 20 min and 4 °C. After washing, the cell pellet was transferred to a fresh 2 L culture of minimal medium (M9 media) supplemented with 25 mg/mL Se-Met, 2% glucose, 0.1 M magnesium sulfate and amino acids, and grown at 37 °C. Protein expression by the cells was induced by the addition of IPTG to a final concentration of 0.5 mM. After 24 h incubation at 37 °C, the cells were harvested by centrifugation at 4,000 *g* for 20 min and 4 °C. The overall purification procedure of the Se-Met substituted _CD_EFhd2 was the same as the native _CD_EFhd2 protein purification procedure. The purified protein was concentrated using an Amicon Ultra-15 30 K (Millipore) and stored in a deep freezer. During purification, the presence of EFhd2 was confirmed by SDS-PAGE.

### Cloning and purification of EFhd2 mutants

To investigate the structural properties of Ca^2+^ binding, we have mutated one acidic residue (E116A for EF1, _CD_EFhd2^EF1^; E152A for EF2, _CD_EFhd2^EF2^) of each EF-hand domain of _CD_EFhd2 to abolish the Ca^2+^ binding ability (see Supplementary Fig. S3). _CD_EFhd2 mutants were accomplished by PCR and site-directed mutagenesis using the _CD_EFhd2 cDNA. All mutants were cloned into a modified pET28a vector (Novagen) containing an N-terminal His_6_-TEV tag. The overall purification procedure of the _CD_EFhd2 mutants was the same as that used for purifying native _CD_EFhd2. To investigate the Ca^2+^-binding affinity or Ca^2+^-dependent actin-binding and -bundling activity, we have mutated one acidic residue of each EF-hand domain of full-length EFhd2 (E116A for EF1, EFhd2^EF1^; E152A for EF2, EFhd2^EF2^) (see Supplementary Fig. S3). Point mutations (EFhd2^EF1^, EFhd2^EF2^) were accomplished by PCR and site-directed mutagenesis using the full-length EFhd2 cDNA. All mutants were cloned into the modified pET21a vector (Novagen) containing an N-terminal His_6_ tag. The overall purification procedure of full-length EFhd2 mutants was the same as that used to purify native full-length EFhd2.

### Crystallisation, data collection, and structure determination

To crystallise native and selenomethionine (Se-Met) substituted _CD_EFhd2, initial screening was performed by the sitting-drop vapour-diffusion method in a 96-well INTELLI-PLATE (Art Robbins Ins.) using the Crystal Screen, Index, SaltRx, MembFac, Natrix, Crystal Screen Lite and Crystal Screen Cryo (Hampton Research). Drops were prepared by mixing 0.5 μL of the protein and 0.5 μL reservoir solutions. Initial crystals of native _CD_EFhd2 were observed in 0.1 M Tris-HCl (pH 8.5), 0.2 M Na-acetate, and 30% (w/v) PEG 4000, and Se-Met substituted _CD_EFhd2 crystals were observed in 0.1 M Tris-HCl (pH 8.5), 0.165 M Na-acetate and 25% (w/v) PEG 4000. Final native and Se-Met substituted _CD_EFhd2 crystals were obtained using a well solution of 0.1 M Tris-HCl (pH 8.5), 23% (w/v) PEG 4000 and 0.16 M Na-acetate, and 0.1 M Tris-HCl (pH 8.5), 0.165 M Na-acetate, 23.5% (w/v) PEG 4000 and 3% (v/v) dioxane, respectively. In addition, crystallisation of _CD_EFhd2^EF1^ and _CD_EFhd2^EF2^ was performed by the sitting-drop vapour-diffusion method in a 96-well INTELLI-PLATE (Art Robbins Ins.) using the Crystal Screen, Index, SaltRx, MembFac, Natrix, Crystal Screen Lite and Crystal Screen Cryo (Hampton Research), and PEGs Suite (Qiagen). Drops were prepared by mixing 0.5 μL of the protein and 0.5 μL reservoir solutions. Crystals of _CD_EFhd2^EF1^ and _CD_EFhd2^EF2^ were observed in 0.1 M Tris-HCl (pH 8.5) and 32% (w/v) PEG 2000. All _CD_EFhd2 crystals were cryoprotected by soaking them for 10 min in mother liquor containing an additional 15% (v/v) glycerol before flash freezing in a stream of nitrogen gas at 95 K. Native and MAD data sets were collected on beamline 7A at the Pohang Accelerator Laboratory (Pohang, Korea). Raw data integration and scaling were performed with the HKL2000^62^. Both the native and Se-Met substituted _CD_EFhd2 were crystallised in the orthorhombic form and space group *P*2_1_2_1_2_1_ and cell dimensions of *a* = 37.3, *b = *50.7, *c* = 53.4 Å. A native data set of 1.85 Å resolution was collected and the MAD dataset of Se-Met substituted protein crystals were collected to 2.10 Å. The crystal contains one molecule in an asymmetric unit with a calculated Matthews coefficient of 1.99 Å^3^/Da and an estimated solvent content of 38.6%^63^. Four out of the expected six Se sites in the asymmetric unit were found using the program SOLVE^64^ using 2.10 Å resolution data yielding phases with a figure of merit of 0.51. Refinement was performed with PHENIX^65^ and manual rebuilding was performed using the COOT program^63^. Cycles of group and individual B-factor refinement were performed with PHENIX^65^. In the last step of the refinement, 117 water and two Ca^2+^ ions were added. A final crystallographic *R*-value of 16.7% (*R*_free_ = 20.2%) was obtained. The N-terminus residues from 70 to 79 of the PxxP motif were poorly defined in the electron density maps owing to disorder in the crystal lattice. Therefore, we could observe the structure of the predicted PxxP motif (residues 80–90) at the N-terminus, two EF-hand domains (residues 91–163) and the connecting short LM α-helix (residues 170–177) region at the C-terminus (Fig. 1). _CD_EFhd2^EF1^ and _CD_EFhd2^EF2^ datasets were collected at beamline 5C at the Pohang Accelerator Laboratory to 1.95 Å and 1.94 Å, respectively. Both the _CD_EFhd2^EF1^ and _CD_EFhd2^EF2^ structures had the space group *P*2_1_2_1_2_1_ and cell dimensions of *a* = 36.3, *b* = 51.5, *c* = 53.6 Å and *a = *35.6, *b = *52.1, *c = *55.3 Å, respectively. Raw data integration and scaling were performed with HKL2000^62^. The Matthews coefficient for _CD_EFhd2^EF1^ and _CD_EFhd2^EF2^ was calculated as 1.84 and 1.88 Å^3^/Da, respectively, which corresponds to a solvent content of 33.0 and 34.7% assuming one molecule in the asymmetric unit^63^. Initial automatic model building was performed with AutoMR. The model was then refined in cyclic rounds of manual model building in COOT with refinement using PHENIX^65^,^66^. Refinement of _CD_EFhd2^EF1^ and _CD_EFhd2^EF2^ was performed using PHENIX to *R*_work_ = 18.2% and *R*_free_ = 20.7%, and *R*_work_ = 17.9% and *R*_free_ = 20.7%, respectively. All structures of the _CD_EFhd2 mutants were solved by molecular replacement using the refined native _CD_EFhd2 structure and molecular graphics were created using PyMol^67^. The refinement statistics are given in Table 1.

### NMR Spectroscopy

For NMR experiments, the _CD_EFhd2-expressing cells were grown in M9 medium containing ^15^N ammonium chloride and ^13^C glucose as the sole nitrogen and carbon sources, respectively. The overall purification procedure followed the approach used to purify the native protein. To remove pre-bound Ca^2+^, proteins were treated with 25-fold excess EGTA and then dialysed extensively against buffer with or without CaCl_2_. During the purification process, 5 mM 3-[(3-cholamidopropyl)dimethylammonio]-1-propanesulfonate was added to the buffer to retain protein stability. The Ca^2+^-dependent structural changes to _CD_EFhd2 were monitored by resonance perturbations in the two-dimensional (2D) ^1^H-^15^N HSQC spectra. NMR data were recorded on a Bruker Avance 800 spectrometer at 25 °C. Data were processed with NMRPipe^68^ and analysed with SPARKY program (Goddard TD and Kneller DG, SPARKY 3, University of California, San Francisco). The magnitude of the ^1^H-^15^N chemical shift differences (∆δ, ppm) were calculated using the equation: ∆δ = {(δH^2^) + 0.2x(δN^2^)}^1/2^, where δH and δN are changes to the proton (^1^H) and nitrogen (^15^N) chemical shift perturbation, respectively. CSPs for peaks that disappeared upon addition of Ca^2+^ are set to 1 ppm. We considered CSP to be significant if ∆δ ≥ 0.2 ppm.

### Measurement of Ca^2+^-binding affinity using ITC

Since the full-length EFhd2 (residues 1–240) was more stable than _CD_EFhd2 in the absence of Ca^2+^, full-length EFhd2 mutants (EFhd2^E116A^, EFhd2^E152A^) were used to measure Ca^2+^-binding affinities of EFhd2. Protein samples were treated initially with 25-fold excess EGTA and EDTA for >20 h at 4 °C to remove pre-bound metal ions. We dialysed extensively against buffer (50 mM Tris-HCl (pH 8.5) and 20 mM NaCl) for 48 h at 4 °C and changing the buffer every 12 h. To measure the residual Ca^2+^ concentration after the dialysis step, we used quantitative fluorescence measurement using the Ca^2+^-indicator fura-2 (non acetoxymethyl ester (AM) form, Molecular Probes, Eugene, OR) (see Supplementary Fig. S9). For determining the intensity of fura-2 at various Ca^2+^ concentrations, we prepared standard solutions refer to the method of Kong *et al*.^69^. After the dialysis process, EFhd2 mutants (EFhd2^EF1^, EFhd2^EF2^) (5 μM) were mixed with 10 μM fura-2. Fluorescence spectra of standard solutions and EFhd2 mutants were collected using a FlexStation Ш (Molecular Devices) at room temperature (excitation wavelength: 280 nm to 460 nm, emission wavelength: 510 nm, slit: 4 nm). The residual Ca^2+^ concentration used for the ITC measurement was around the 1 nM range, indicating that our dialysis process was sufficient to measure Ca^2+^ binding affinity using the ITC experiment. The protein sample (70 and 150 μM for EFhd2^EF1^ and EFhd2^EF2^, respectively) was titrated with 30 injections of ligand (10 μL) in a VP-ITC calorimeter (MicroCal). The ligand solution (0.6 and 1.2 mM Ca^2+^ for EFhd2^EF1^ and EFhd2^EF2^, respectively) was prepared in the same buffer. All measurements were conducted at 25 °C, and binding isotherms were analysed using Origin software supplied with the calorimeter.

### Protein stability measured using a heat aggregation assay

To measure the stability of EFhd2 in a Ca^2+^-dependent manner, the half aggregation temperature of native _CD_EFhd2, _CD_EFhd2^EF1^, and _CD_EFhd2^EF2^ were determined spectrophotometrically. The protein solution contained 5 mM Tris-HCl (pH 8.0), 1 mM CaCl_2_ and 250 μM of protein in a final volume of 4.0 mL. The temperature was increased at the rate of 4 °C per 90 s. Turbidity was monitored by the absorption at 470 nm and room temperature using an ultraviolet-visible spectrometer (Ultrospec 2000; Pharmacia Biotech).

### Ensemble refinement of _CD_EFhd2, _CD_EFhd2^EF1^ and _CD_EFhd2^EF2^

To evaluate structural dynamics at the atomic level, we performed ensemble refinement using the Phenix.ensemble refinement^31^ for _CD_EFhd2, _CD_EFhd2^EF1^ and _CD_EFhd2^EF2^. Harmonic restraints were applied for all amino acids with visible electron density at a level of 1σ in the 2m*F*o-D*F*c electron density map using parameters slack = 1.0 and weight = 0.001.

### Protein preparation and the crosslinking experiment of cysteine mutants within the EFhd2 coiled-coil domain (residues 199–240)

To determine whether the C-terminal coiled-coil domain formed a dimer by parallel or antiparallel coiled-coil interaction, we designed recombinant fragments of the coiled-coil domain (residues 199–240) with Cys mutations at the N-terminus (CC1) or C-terminus (CC2) of the coiled-coil domain. The sequence for CC1 starts with CysGlyGly at the N-terminus, whereas for CC2 the C-terminus ends with GlyGlyCys. CC1 and CC2 clones were PCR amplified from the cDNA of the coiled-coil domain of EFhd2. CC1 was subcloned into pGEX-4T-1 and the glutathione S-transferase (GST) tag at the N-terminus was removed by thrombin treatment during the purification process. In addition, CC2 was subcloned into a modified pET-21a vector (Novagen) containing an N-terminal His_6_ tag. The purification procedure was the same as those used for the other EFhd2 proteins. CC1 and CC2 proteins were analysed by SDS-PAGE under reducing and non-reducing conditions to identify the disulfide bond between Cys residues that mediate dimerisation.

### *In vitro* actin-binding and -bundling assay

F-Actin binding (co-sedimentation) and bundling assays were performed as reported^26^. In brief, non-muscle actin derived from human platelets was purchased from Cytoskeleton Inc. (Denver, CO, USA). Actin was mixed in G-buffer (5 mM Tris-HCl, pH 8.0 and 0.5 mM CaCl_2_) to produce an actin stock solution and polymerised in actin polymerisation buffer (0.2 mM Tris-HCl, pH 8.0, 100 mM KCl, 2 mM MgCl_2_ and 0.5 mM ATP) at room temperature for 1 h and then incubated with EFhd2 or its mutants from 5 min to 1 h at room temperature. Actin filaments with bound proteins were pelleted by centrifugation at 100,000 *g* for 2 h at room temperature (for the F-actin binding assay) or 15,000 *g* for 10 min at room temperature (for the F-actin bundling assay). BSA and α-actinin were used as a negative and positive control, respectively. Equal amounts of pellet and supernatant were resolved by SDS-PAGE and proteins were visualised by Coomassie Blue staining. The percentage of actin in the supernatant (S) and pellet (P) was quantified by densitometry using ImageJ 1.44p.

## Additional Information

**Accession codes:** Atomic coordinates and structure factors of _CD_EFhd2, _CD_EFhd2^EF1^, and _CD_EFhd2^EF2^ have been deposited in the RCSB Protein Data Bank with accession codes 5I2L, 5I2O, and 5I2Q.

**How to cite this article**: Park, K. R. *et al*. Structural implications of Ca^2+^-dependent actin-bundling function of human EFhd2/Swiprosin-1. *Sci. Rep.* **6**, 39095; doi: 10.1038/srep39095 (2016).

**Publisher's note:** Springer Nature remains neutral with regard to jurisdictional claims in published maps and institutional affiliations.

## Supplementary Material

Supplementary Information

## Figures and Tables

**Table 1:** Data collection and refinement statistics.

Data Collection	_CD_EFhd2					_CD_EFhd2^EF1^	_CD_EFhd2^EF2^
	Native	SeMet derivative					
		Inflection	Peak	H-Remote	L-Remote		
X-ray source^a^	PAL-7A					PAL-5C	
Wavelength (Å)	1.0000	0.9796	0.9793	0.9716	0.9873	0.9793	0.9793
Resolution (Å)	50–1.85	50–2.10				50–1.95	50–1.94
Space group	*P*2_1_2_1_2_1_	*P*2_1_2_1_2_1_				*P*2_1_2_1_2_1_	
Unit-cell parameters (Å)	*a* = 37.3*b* = 50.7*c* = 53.4	*a* = 42.1*b* = 50.4*c* = 68.1	*a* = 42.2*b* = 50.5*c* = 68.2	*a* = 42.1*b* = 50.4*c* = 68.0	*a* = 42.3*b* = 50.4*c* = 68.1	*a* = 36.3*b* = 51.5*c* = 53.6	*a* = 35.6*b* = 52.1*c* = 55.3
No. of observed reflections	85,596	132,152	118,758	127,813	108,283	81,998	74,173
No. of unique reflections	8,999	8,469	8,417	8,305	8,506	7,706	7,948
Completeness (%)	99.3 (97.3)	99.7 (95.0)	99.7 (96.2)	98.8 (87.6)	99.9 (97.9)	98.7 (78.7)	98.8 (98.7)
*R*_merge_^b^ (%)	8.3 (41.2)	12.4 (40.8)	14 (43.2)	15.4 (47.5)	10.5 (36.7)	6.2 (38.3)	4.7 (27.1)
Mean I/σ (I)	13.1 (9.9)	8.2 (10.4)	8.0 (7.3)	6.3 (6.1)	8.5 (10.2)	9.8 (7.5)	15.4 (6.1)
Multiplicity	9.4 (9.7)	14.8 (12.1)	13.3 (11.2)	14.5 (11.6)	12.1 (10.5)	10.6 (7.7)	9.3 (8.7)
**Refinement statistics**							
Resolution (Å)	50–1.85					50–1.95	50–1.93
*R*_work_/*R*_free_^c^ (%)	16.7/20.2					18.2/20.7	17.9/20.7
Protein	857					850	849
Ca^2+^	2					1	1
Water	119					44	61
Bond length (Å)	0.010					0.015	0.009
Bond angle (°)	1.27					1.45	1.02
Most favored regions (%)	99.04					98.98	97.09
Allowed regions (%)	0.96					1.02	2.91
PDB code	5I2L					5I2O	5I2Q

^a^Beamline 5C and 7A at Pohang Accelerator Laboratory (PAL) in South Korea.

^b^*R*_merge_ = ∑_*h*_ ∑_*i*_│I(*h*)_*i*_−‹I(*h*)›│/∑_*h*_ ∑_*i*_I(*h*)_*i*_, where I(*h*) is the intensity of reflection of *h*, ∑_*h*_ is the sum over all reflections, and ∑_*i*_ is the sum over *i* measurements of reflection *h*.

^c^*R*_work_ = Σ_*hkl*_||F_*o*_|–|F_*c*_||/Σ_*hkl*_|F_*o*_|; *R*_free_ is the *R* value calculated for 5% of the data set not included in the refinement.
