# Supplementary material for: Structural implications of Ca2+-dependent actin-bundling function of human EFhd2/Swiprosin-1
Source: Sci Rep. 2016 Dec 15;6:39095. doi: 10.1038/srep39095 (PMC5156911; doi:10.1038/srep39095)
Supplement: Supplementary Information [file srep39095-s1.pdf]

Supplementary Information

Structural implications of Ca^{2+}-dependent actin-bundling function of human EFhd2/Swiprosin-1

Kyoung Ryoung Park[1,2*], Min-Sung Kwon[1*], Jun Yop An[1,2], Jung-Gyu Lee[1,2], Hyung-Seop Youn[1,2], Youngjin Lee[1,2], Jung Youn Kang[1,2], Tae Gyun Kim[1,2†], Jia Jia Lim[1,2], Jeong Soon Park[3], Sung Haeng Lee[4], Woo Keun Song[1], Hae-Kap Cheong[3], Chang-Duk Jun[1] & Soo Hyun Eom[1,2]

[1]School of Life Science, Gwangju Institute of Science and Technology (GIST), 123 Cheomdangwagi-ro, Buk-gu, Gwangju 61005, Republic of Korea. [2]Steitz Center for Structural Biology and Department of Chemistry, Gwangju Institute of Science and Technology (GIST), 123 Cheomdangwagi-ro, Buk-gu, Gwangju 61005, Republic of Korea. [3]Protein Structure Group, Korea Basic Science Institute, 162 Yeongudanji-Ro, Ochang 363-883, Republic of Korea. [4]Department of Cellular and Molecular Medicine, Chosun University School of Medicine, 375 Seosuk-dong, Dong-gu, Gwangju 501-759, Republic of Kore

[*]These authors contributed equally to this work.

[†] Current address: Molecular Cryo-Electron Microscopy Unit, Okinawa Institute of Science and Technology Graduate University, 1919-1 Tancha, Onna, Kunigami, Okinawa 904-0495, Japan.

Correspondence and requests for materials should be addressed to S.H.E. (email: eom@gist.ac.kr)

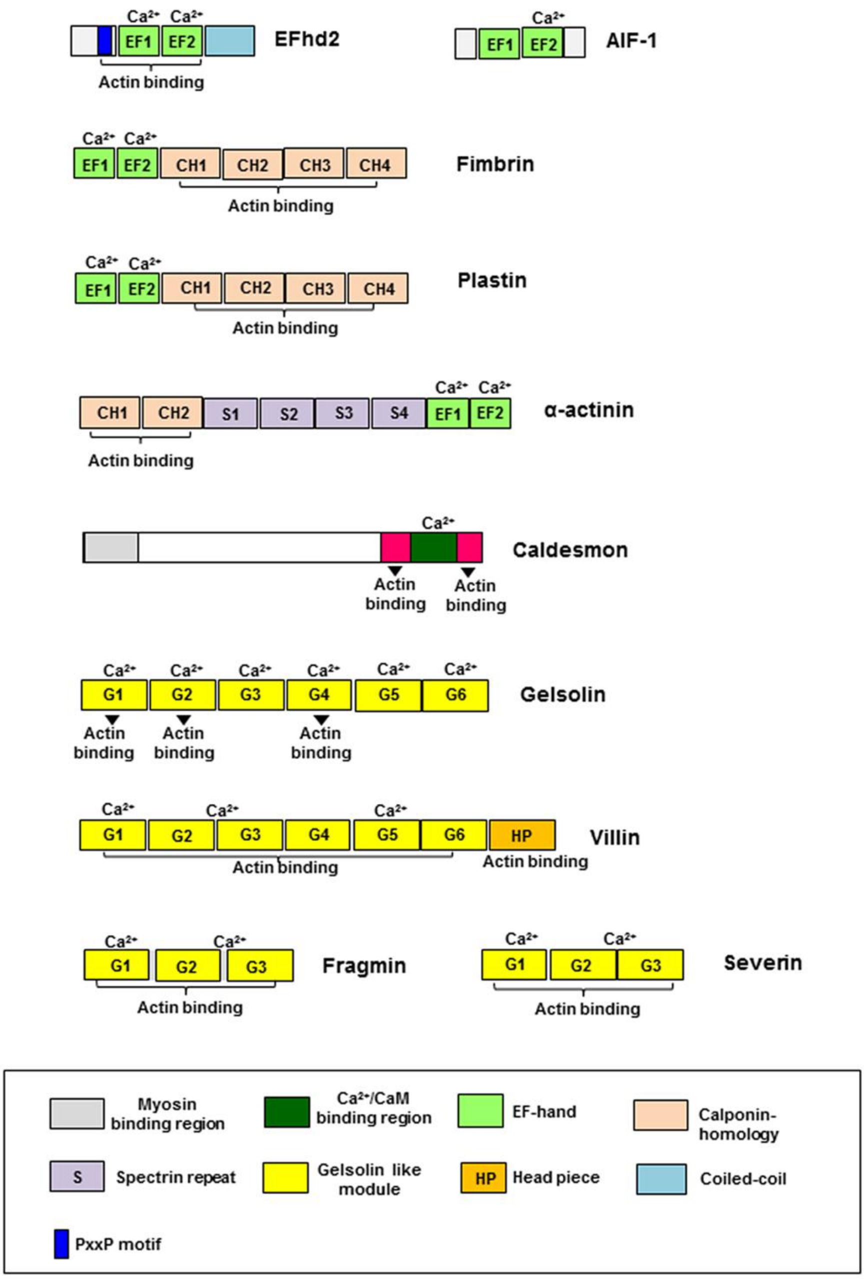

Supplementary Figure S1. Domain structures of Ca^{2+}-related actin-binding proteins. Schematic showing the domain organisation of Ca^{2+}-related actin-binding proteins. CH domains and gelsolin repeat modules function as actin-binding regions. EF-hands and the CaM-binding region function as Ca^{2+}-binding regions; however, EF1 of AIF-1 does not bind to Ca^{2+} and the actin-binding site of AIF-1 is unclear.

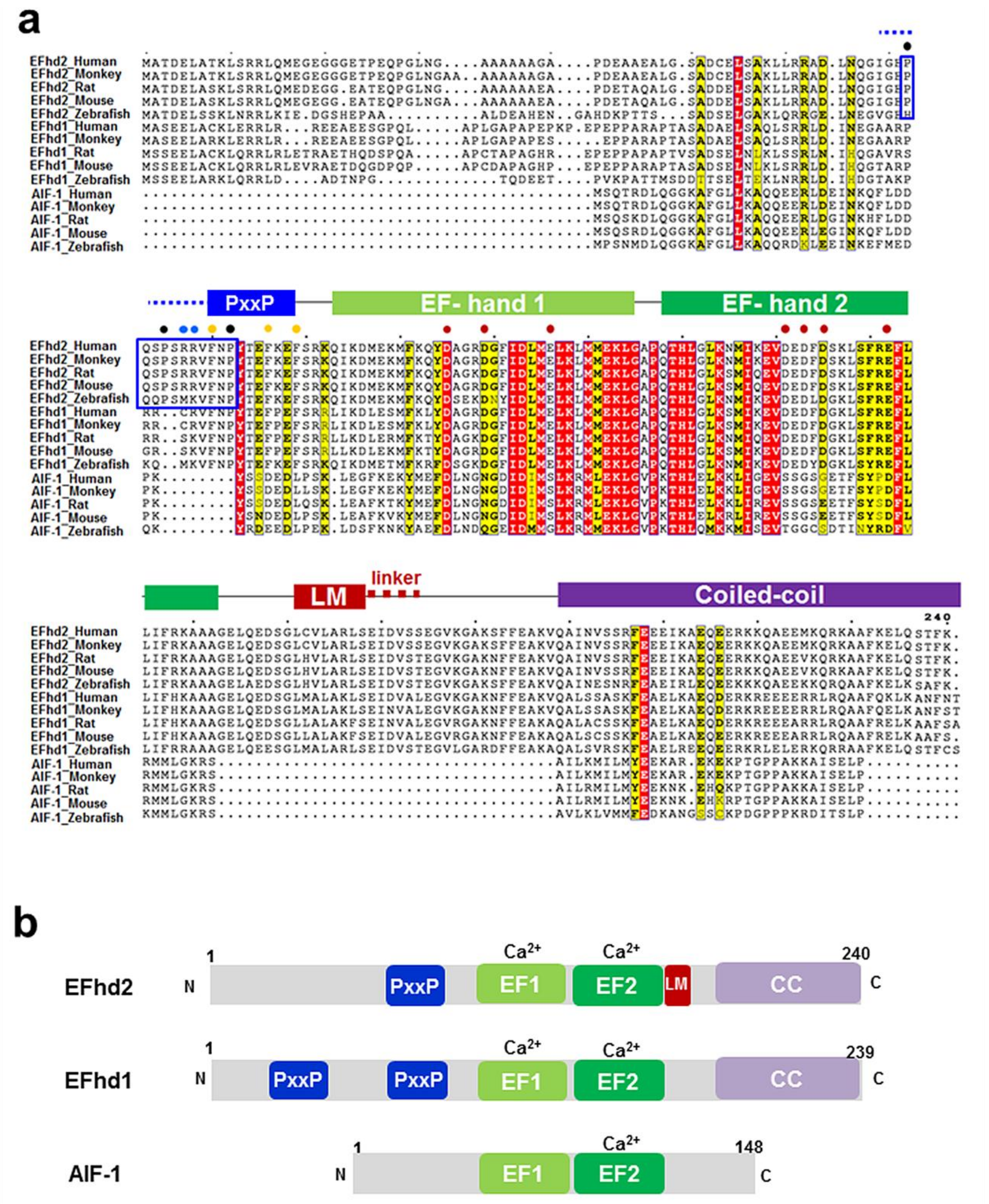

Supplementary Figure S2. Comparison of the domain organisation of EFhd2, EFhd1 and AIF-1. The multiple sequence alignment was carried out using the ENDscript server[71]. PxxP : proline-rich region; EF: EF-hands; LM: ligand mimic; CC: coiled-coil. The disordered region of the PX domain in the crystal structure of $_{CD}$EFhd2 is shown by the blue dashed line.

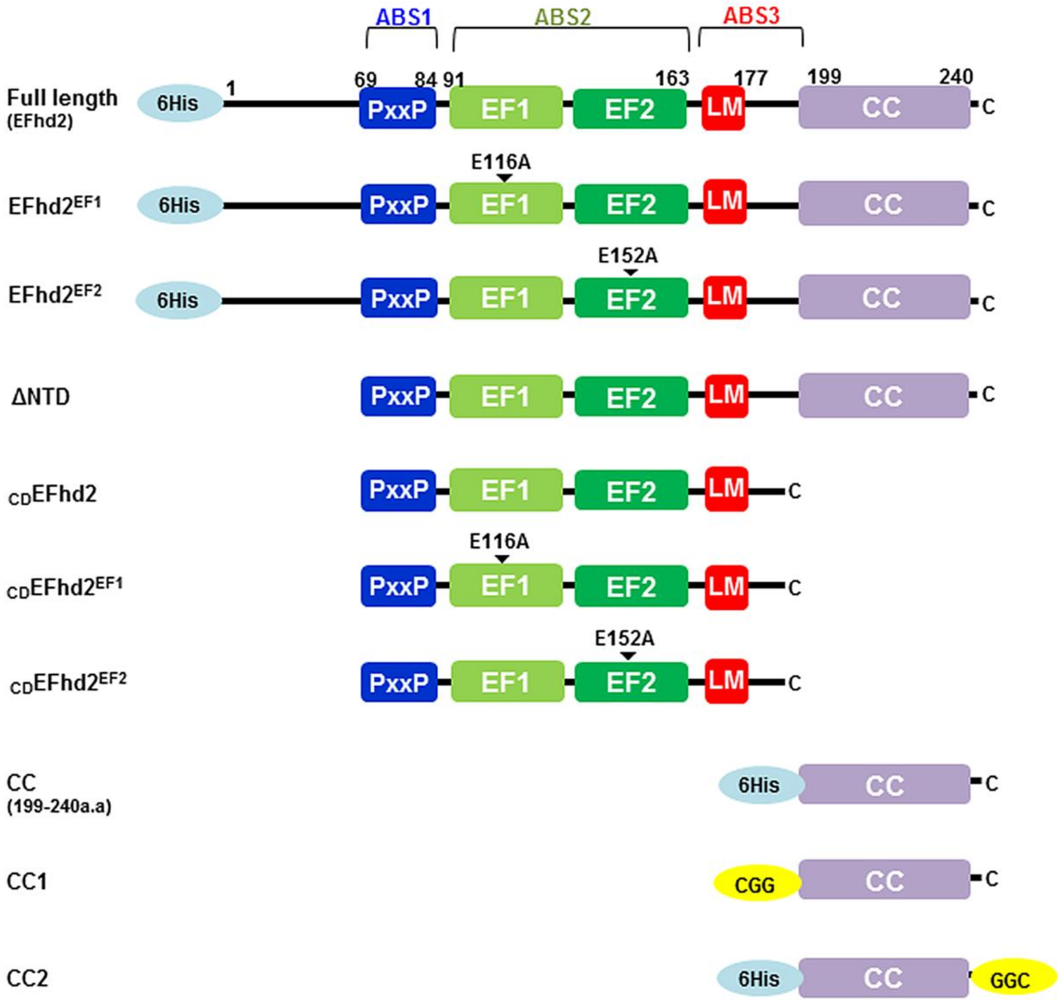

Supplementary Figure S3. Schematic of the EFhd2 constructs.

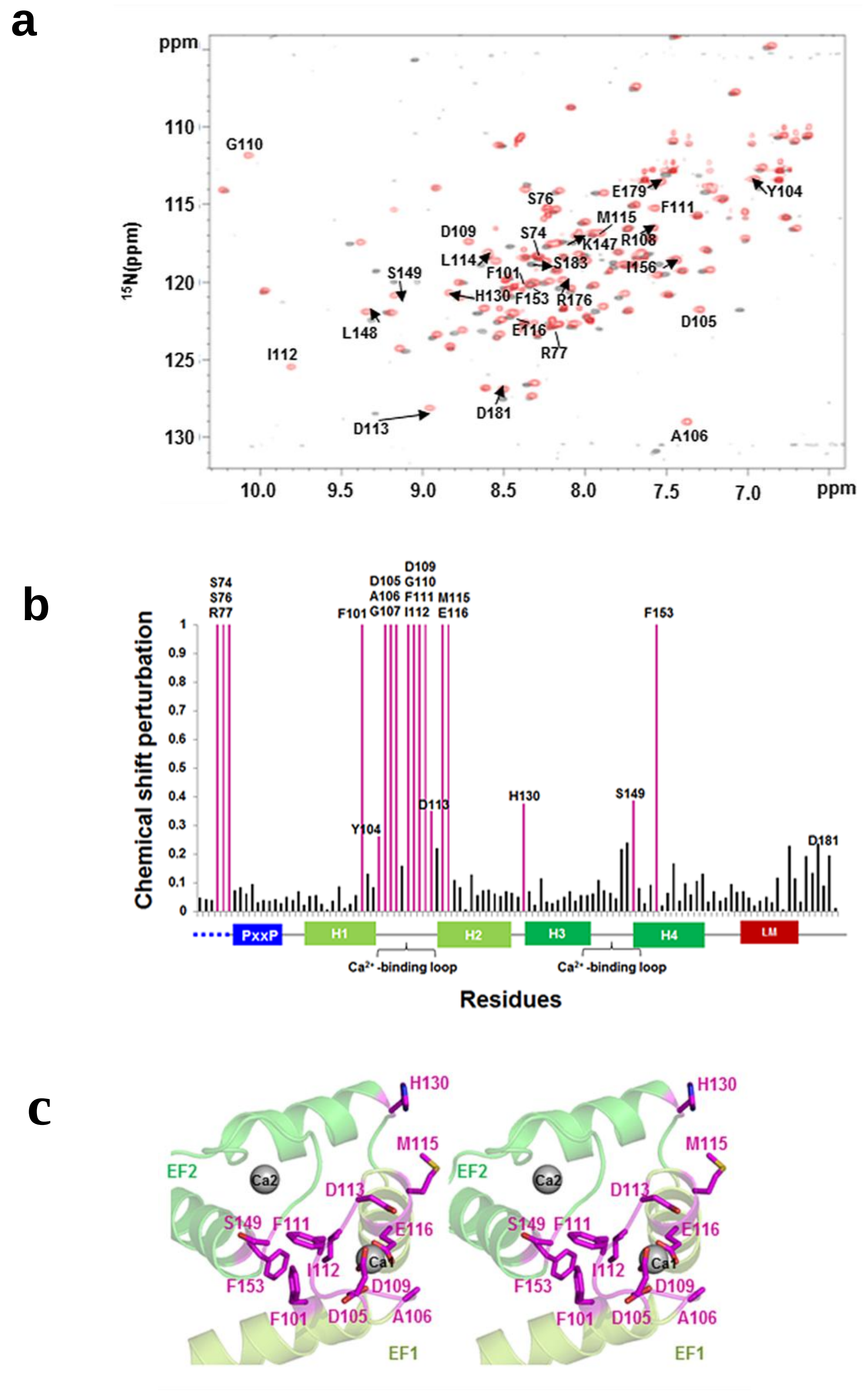

Supplementary Figure S4. Structural characteristics of $_{CD}$EFhd2 in the absence of Ca^{2+}.

(**a**) 2D ^{1}H-^{15}N HSQC spectra of $_{CD}$EFhd2 in the presence or absence of Ca^{2+}. The chemical

shifts are monitored in ^{1}H, ^{15}N correlation spectra. The resonances of $_{CD}$EFhd2 in the presence of Ca^{2+} are shown in red and resonances arising from $_{CD}$EFhd2 in the absence of Ca^{2+} are shown in black. Residues disappearing (set $\Delta\delta = 1$ ppm) or showing significant changes in chemical shifts ($\Delta\delta \geq 0.2$ ppm) upon addition of Ca^{2+} are labelled. (**b**) Chemical shift perturbation (CSP) analysis of $_{CD}$EFhd2 in the presence or absence of Ca^{2+}. The disordered region of the PxxP motif in the crystal structure of $_{CD}$EFhd2 is shown by the blue dashed line. (**c**) Representation of the perturbed residues (shown as stick in magenta) in the crystal structure of $_{CD}$EFhd2.

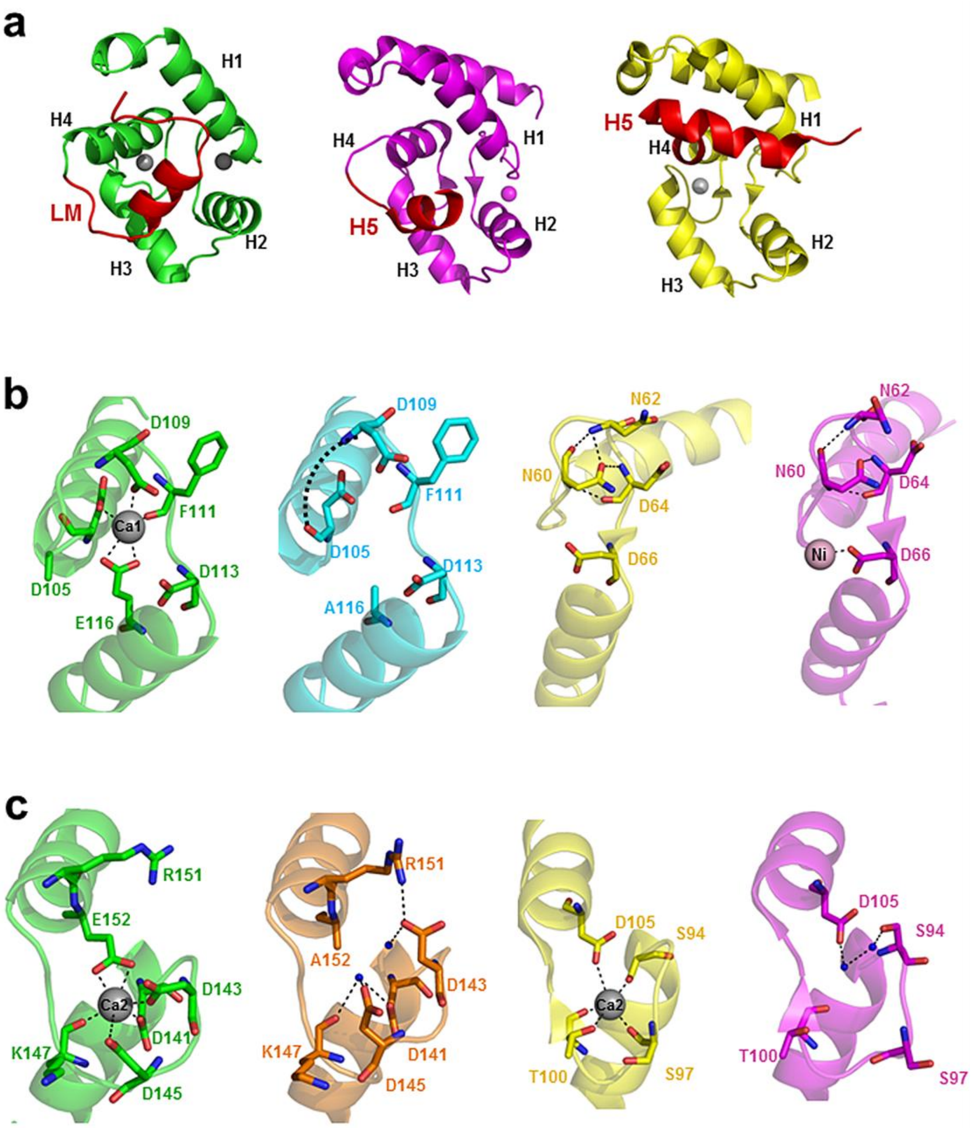

Supplementary Figure S5. Structural comparison between $_{CD}$EFhd2 with homologous protein, AIF-1. (**a**) Structures of Ca^{2+}-bound $_{CD}$EFhd2 (green), Ca^{2+}-free AIF-1 (PDB ID: 2D58, magenta) and Ca^{2+}-bound AIF-1 (PDB ID: 1WY9, yellow)[35]. Grey and magenta spheres indicate calcium and nickel ions, respectively. (**b**) Ca^{2+}-binding loop of EF1 of the Ca^{2+}-bound $_{CD}$EFhd2 (green), $_{CD}$EFhd2^{EF1}(cyan), Ca^{2+}-bound AIF-1 (yellow) and Ca^{2+}-free AIF-1 (magenta).（c）Ca^{2+}-binding loop of EF2 of the Ca^{2+}-bound $_{CD}$EFhd2 (green), $_{CD}$EFhd2^{EF2} (orange), Ca^{2+}-bound AIF-1 (yellow) and Ca^{2+}-free AIF-1 (magenta). Blue spheres indicate water molecules.

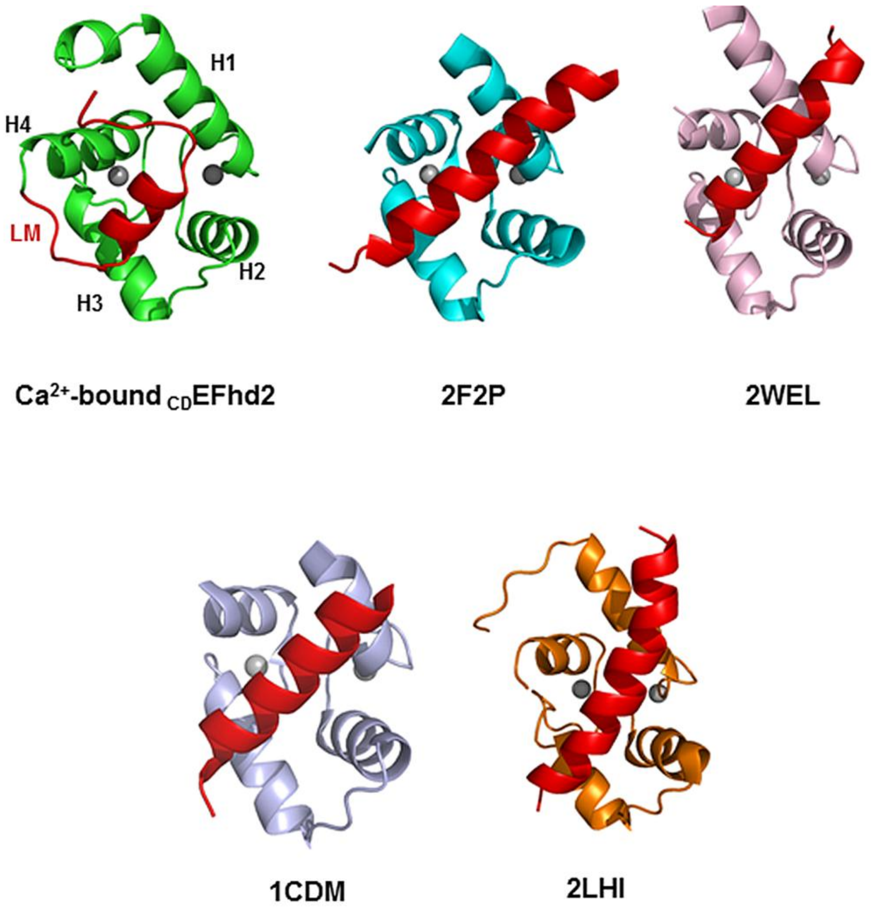

Supplementary Figure S6. Structural comparison between $_{CD}$EFhd2 and EF-hand-containing proteins. Structure of Ca^{2+}-bound $_{CD}$EFhd2 (green) and CaM with the target peptide complex (PDB ID: 2F2P, 2WEL, 1CDM and 2LHI)[38-41]. Red ribbon diagram shows the LM of the $_{CD}$EFhd2 and target peptides of CaM. Silver spheres indicate Ca^{2+} ions. A search for structures similar to $_{CD}$EFhd2 using the Dali program[37] gave nearly 100 Ca^{2+}-bound EF-hands (>10 for Z-scores and < 3.0 Å in RMSD), in which most of the EF-hand matches were calmodulin (CaM) and troponin C (TnC). In addition, the structure of $_{CD}$EFhd2 fits well with those of Ca^{2+}-peptide-CaM such as PDB IDs 2F2P (Z-score = 11.5, RMSD = 1.6 Å for 79 Cα atoms), 2WEL (Z-score = 11.2, RMSD =1.74 Å for 79 Cα atoms), 1CDM (Z-score = 10.6, RMSD = 1.7 Å for 73 Cα atoms) and 2LHI (Z-score = 10.5, RMSD = 1.87 Å for 81 Cα atoms).

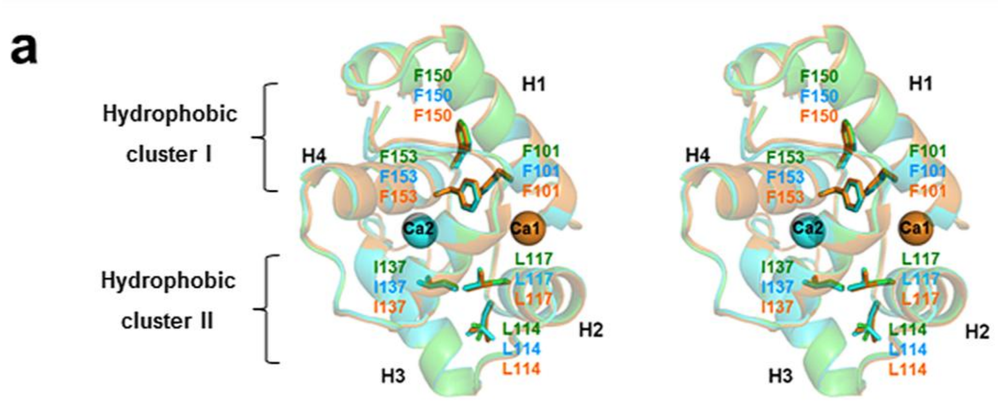

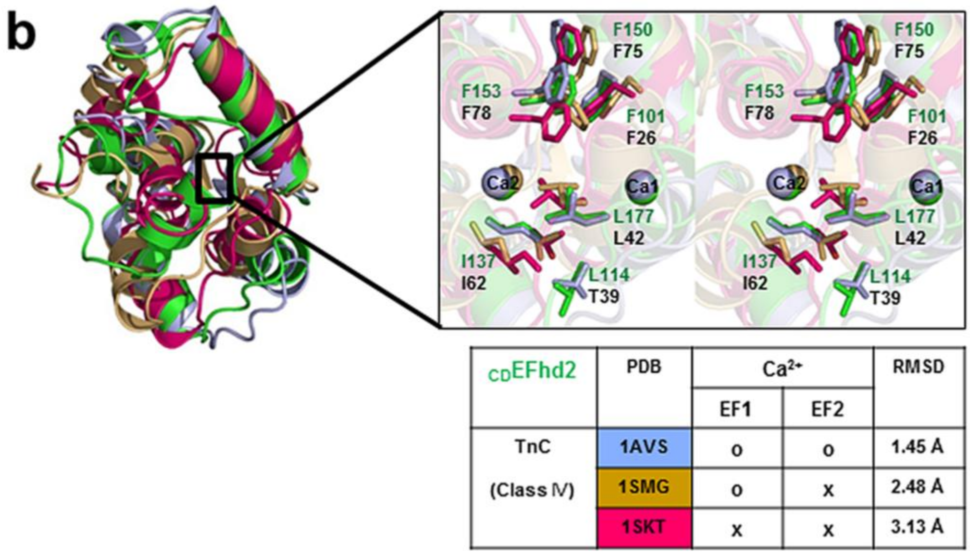

$_{CD}$EFhd2	PDB	Ca^{2+}		RMSD
		EF1	EF2	
TnC (Class IV)	1AVS	o	o	1.45 Å
	1SMG	o	x	2.48 Å
	1SKT	x	x	3.13 Å

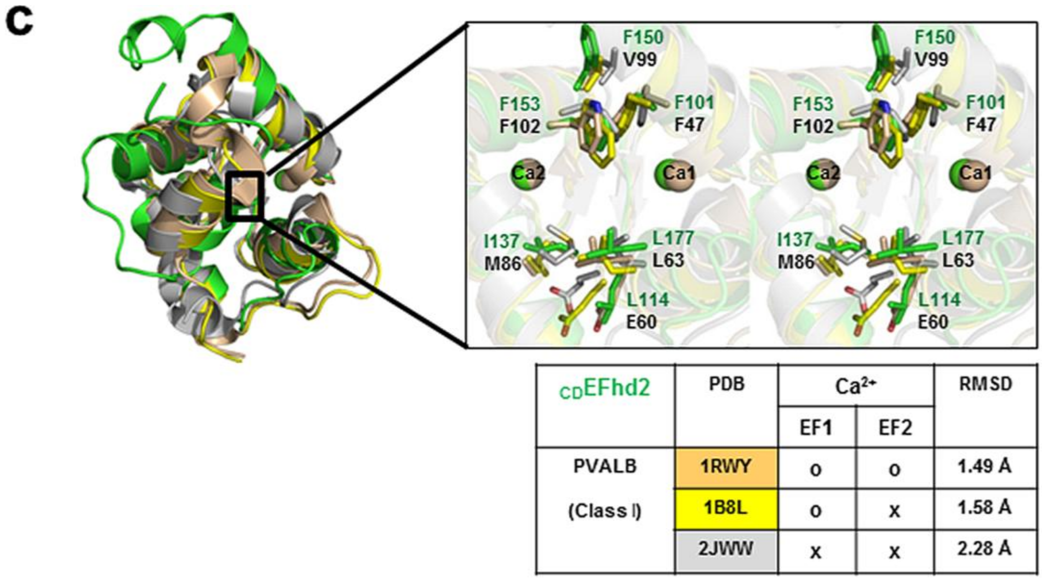

$_{CD}$EFhd2	PDB	Ca^{2+}		RMSD
		EF1	EF2	
PVALB (Class I)	1RWY	o	o	1.49 Å
	1B8L	o	x	1.58 Å
	2JWW	x	x	2.28 Å

Supplementary Figure S7. Hydrophobic clusters of EFhd2. **(a)** Hydrophobic residues within hydrophobic cluster of the Ca^{2+}-bound $_{CD}$EFhd2 (green), $_{CD}$EFhd2^{EF1} (cyan), and $_{CD}$EFhd2^{EF2}

(orange) **(b)** Structural superposition of Ca^{2+}-bound $_{CD}$EFhd2 (green) and TnC (1AVS, Ca^{2+}-bound form, RMSD = 1.45 Å for 77 Cα atoms; 1smg, one Ca^{2+}-bound form, RMSD = 2.48 Å for 72 Cα atoms; 1SKT, apo-form, RMSD = 3.13 Å for 64 Cα atoms)[49-51]. **(b)** Structural superposition of Ca^{2+}-bound $_{CD}$EFhd2 (green) and PVALB (1RWY, Ca^{2+}-bound form, RMSD = 1.49 Å for 67 Cα atoms; 1B8L, one Ca^{2+}-bound form, RMSD = 1.58 Å for 62 Cα atoms; 2JWW, apo-form, RMSD = 2.28 Å for 67 Cα atoms)[52-54]. Residues of Ca-bound and PVALB are labelled green and black, respectively.

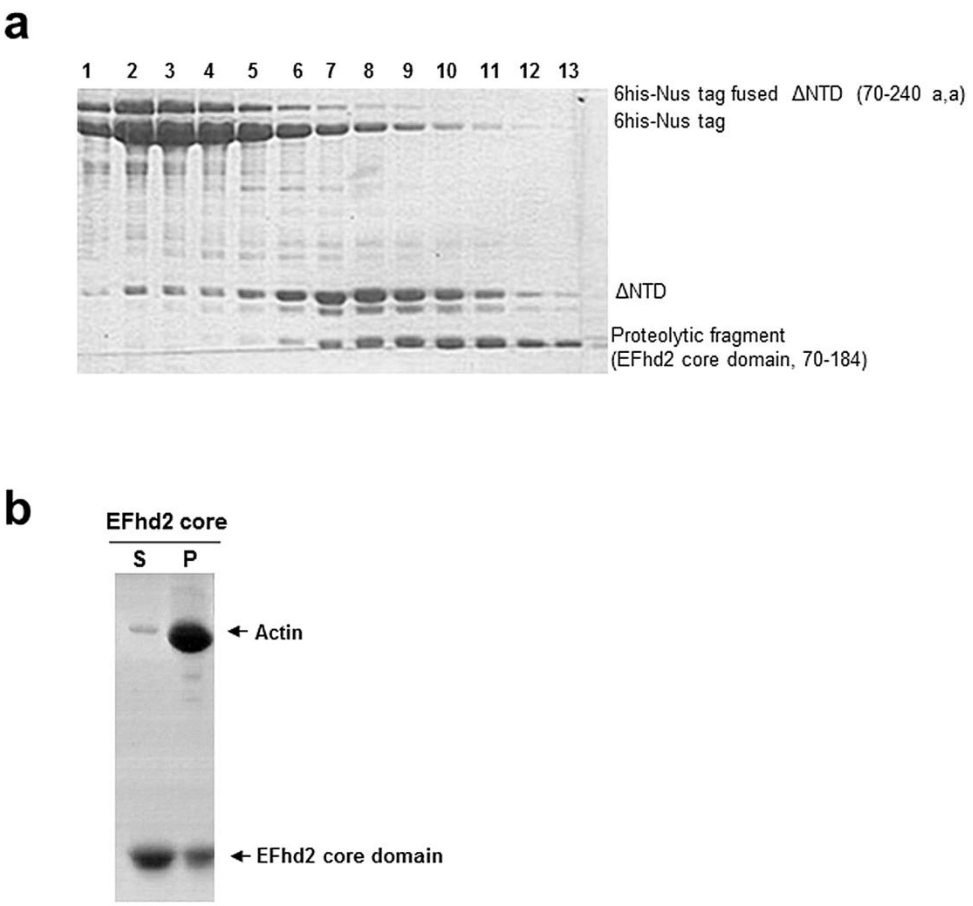

Supplementary Figure S8. Identification of $_{CD}$EFhd2. (**a**) All samples collected during the size exclusion chromatography procedure. Purified ΔNTD (residues 70−240) was incubated with TEV protease for 10 h at 4 °C prior to buffer exchange through size exclusion chromatography. The resulting fragments were resolved by SDS-PAGE. (**b**) Co-sedimentation (actin-binding) assay of $_{CD}$EFhd2. Protein samples (5 μM) were added to pre-polymerised actin (2 μM) in the presence of 1 mM $CaCl_2$.

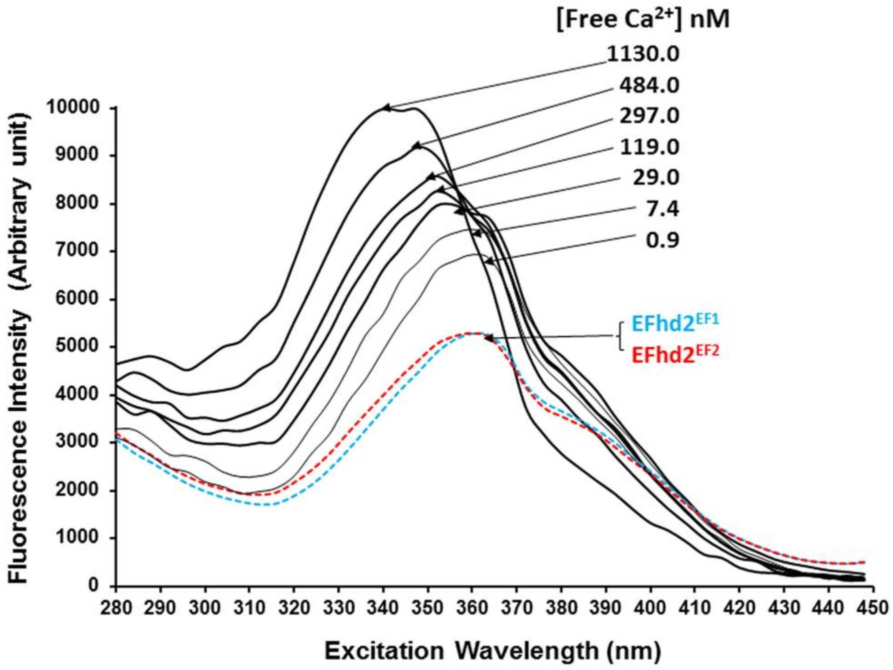

Supplementary Figure S9. Fluorescence spectra of fura-2 in the presence of various concentrations of Ca^{2+} are represented by black lines. Fluorescence spectra of fura-2 mixed with $EFhd2^{EF1}$ and $EFhd2^{EF2}$ is represented by cyan and orange dashed line, respectively. Standard solutions to determining Ca^{2+} concentration were prepared refer to the method of Kong *et* al[69].
